# Development and validation of a prognostic model incorporating tumor thrombus grading for nonmetastatic clear cell renal cell carcinoma with tumor thrombus: A multicohort study

**DOI:** 10.1002/mco2.300

**Published:** 2023-07-20

**Authors:** Le Qu, Hui Chen, Qi Chen, Silun Ge, Aimin Jiang, Nengwang Yu, Yulin Zhou, Michał Kunc, Ye Zhou, Xiang Feng, Wei Zhai, Zhenjie Wu, Miaoxia He, Yaoming Li, Rui Chen, Bo Han, Xing Zeng, Yao Fu, Changwei Ji, Xiang Fan, Guangyuan Zhang, Cheng Zhao, Taile Jing, Chenchen Feng, Hongwei Zhao, Di Sun, Liang Wang, Sheng Tai, Cheng Zhang, Shaohao Chen, Yixun Liu, Haifeng Wang, Jinli Gao, Yufeng Gu, He Miao, Tangliang Zhao, Xiaoming Yi, Chaopeng Tang, Dian Fu, Haowei He, Qiu Rao, Wenquan Zhou, Ning Xu, Gongxian Wang, Chaozhao Liang, Zhiyu Liu, Dan Xia, Xiongbing Zu, Ming Chen, Hongqian Guo, Weijun Qin, Zhe Wang, Wei Xue, Benkang Shi, Shaogang Wang, Junhua Zheng, Cheng Chen, Łukasz Zapała, Jingping Ge, Linhui Wang

**Affiliations:** ^1^ Department of Urology Jinling Hospital Affiliated Hospital of Medical School Nanjing University Nanjing Jiangsu China; ^2^ Department of Urology Jinling Hospital Jinling School of Clinical Medicine Nanjing Medical University Nanjing Jiangsu China; ^3^ Department of Pathology Jinling Hospital Affiliated Hospital of Medical School Nanjing University Nanjing Jiangsu China; ^4^ Department of Health Statistics Naval Medical University Shanghai China; ^5^ Department of Urology Changhai Hospital Naval Medical University Shanghai China; ^6^ Department of Urology Qilu Hospital of Shandong University Jinan Shandong China; ^7^ Department of Pathomorphology Medical University of Gdańsk Gdańsk Poland; ^8^ Department of Urology Renji Hospital School of Medicine Shanghai Jiao Tong University Shanghai Shanghai China; ^9^ Department of Pathology Changhai Hospital Naval Medical University Shanghai China; ^10^ Department of Urology Daping Hospital Army Medical University Chongqing China; ^11^ Department of Pathology Qilu Hospital of Shandong University Jinan Shandong China; ^12^ Department of Urology Tongji Hospital Tongji Medical College Huazhong University of Science and Technology Wuhan Hubei China; ^13^ Department of Pathology Drum Tower Hospital Clinical School of Medical College Nanjing University Nanjing Jiangsu China; ^14^ Department of Urology Drum Tower Hospital Clinical School of Medical College Nanjing University Nanjing Jiangsu China; ^15^ Department of Pathology Zhongda Hospital Southeast University Nanjing Jiangsu China; ^16^ Department of Urology Zhongda Hospital Southeast University Nanjing Jiangsu China; ^17^ Department of Urology Xiangya Hospital Central South University Changsha Hunan China; ^18^ Department of Urology The First Affiliated Hospital School of Medicine Zhejiang University Hangzhou Zhejiang China; ^19^ Department of Urology Huashan Hospital Fudan University Shanghai China; ^20^ Department of Urology Affiliated Yantai Yuhuangding Hospital Qingdao University Yantai Shandong China; ^21^ Department of Pathology Affiliated Yantai Yuhuangding Hospital Qingdao University Yantai Shandong China; ^22^ Department of Urology The Second Affiliated Hospital of Dalian Medical University Dalian Liaoning China; ^23^ Department of Urology The First Affiliated Hospital of Anhui Medical University Hefei Anhui China; ^24^ Department of Urology The First Affiliated Hospital of Nanchang University Nanchang Jiangxi China; ^25^ Department of Urology Urology Research Institute The First Affiliated Hospital Fujian Medical University Fuzhou Fujian China; ^26^ Department of Urology Anhui Provincial Hospital The First Hospital of the University of Science and Technology of China Hefei Anhui China; ^27^ Department of Urology Shanghai East Hospital Tongji University Shanghai China; ^28^ Department of Pathology Shanghai East Hospital Tongji University Shanghai China; ^29^ Department of Urology Xijing Hospital Fourth Military Medical University Xi'an Shanxi China; ^30^ Department of Pathology Xijing Hospital Fourth Military Medical University Xi'an Shanxi China; ^31^ Department of Medical Oncology Jinling Hospital Affiliated Hospital of Medical School Nanjing University Nanjing Jiangsu China; ^32^ Clinic of General Oncological and Functional Urology Medical University of Warsaw Warsaw Poland

**Keywords:** clear cell renal cell carcinoma, pathological grading, prognostic model, risk stratification, venous tumor thrombus

## Abstract

There is significant variability with respect to the prognosis of nonmetastatic clear cell renal cell carcinoma (ccRCC) patients with venous tumor thrombus (VTT). By applying multiregion whole‐exome sequencing on normal‐tumor‐thrombus‐metastasis quadruples from 33 ccRCC patients, we showed that metastases were mainly seeded by VTT (81.8%) rather than primary tumors (PTs). A total of 706 nonmetastatic ccRCC patients with VTT from three independent cohorts were included in this study. C‐index analysis revealed that pathological grading of VTT outperformed other indicators in risk assessment (OS: 0.663 versus 0.501–0.610, 0.667 versus 0.544–0.651, and 0.719 versus 0.511–0.700 for Training, China‐Validation, and Poland‐Validation cohorts, respectively). We constructed a risk predicting model, TT‐GPS score, based on four independent variables: VTT height, VTT grading, perinephric fat invasion, and sarcomatoid differentiation in PT. The TT‐GPS score displayed better discriminatory ability (OS, c‐index: 0.706–0.840, AUC: 0.788–0.874; DFS, c‐index: 0.691–0.717, AUC: 0.771–0.789) than previously reported models in risk assessment. In conclusion, we identified for the first‐time pathological grading of VTT as an unheeded prognostic factor. By incorporating VTT grading, the TT‐GPS score is a promising prognostic tool in predicting the survival of nonmetastatic ccRCC patients with VTT.

## Graphical abstract

Inspired by the metastasis‐seeding potential of VTT in ccRCC, we identified for the first time that pathological grading of VTT could serve as an unheeded prognostic factor. By incorporating VTT grading, TT‐GPS score was constructed, displaying superior discriminatory ability for risk stratification in nonmetastatic ccRCC patients with VTT. This study highlights the significance of introducing VTT grading or TT‐GPS score into routine pathological reports to improve the efficacy of risk assessment.

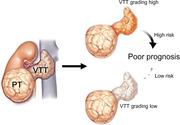

## 1 INTRODUCTION

One biological characteristic of renal cell carcinoma (RCC) is its propensity to extend into the venous system. Venous tumor thrombus (VTT) is observed in 4−10% of newly diagnosed RCC patients,^1^ and surgery remains the mainstay of treatment for these patients.^2^ A successful radical nephrectomy and thrombectomy provides considerable palliation to a proportion of nonmetastatic RCC patients with VTT, sometimes leading to a higher long‐term survival rate.^3^ However, the reported postsurgical survival varies significantly with the 5‐year overall survival (OS) rate ranging from 37.0 to 71.0%.^1^ Hence, accurate risk positioning models are critically needed for these patients.

Thrombus has long been considered as a simple extension of the primary tumor (PT) to the vessel and assumed to have almost the same molecular profiling and histological characteristics as the PT.^4^, ^5^ The TRACERx Renal project gained an insight into the ability of VTT to act as a source of metastatic dissemination with a limited sample size of clear cell renal cell carcinoma (ccRCC) patients.^6^, ^7^ In this study, we tested the evolutionary hypothesis with expanded sample size, which is the largest matched sample size to date.

At present, most prognostic models were developed from a general population of RCC patients, not specific for nonmetastatic RCC patients with VTT.^3^, ^8^, ^9^, ^10^, ^11^ Recently, Abel et al.^12^, ^13^ developed two models with higher predictive accuracy than UISS model and SSIGN score for these patients. However, the reported models only analyzed one feature specific for VTT, the thrombus height. In addition, almost all pathologists and doctors only paid attention to whether there are tumor cells within VTT in common practice, and ignored the possibility that the characteristics of VTT might provide extra information on oncologic outcomes.

Here, we explored whether the multiple characteristics of VTT might hold untapped potential for ccRCC risk assessment. To the best of our knowledge, this is the first study to report the role of VTT grading as a prognostic biomarker in a large multi‐institutional cohort of nonmetastatic ccRCC patients with VTT. This study also highlights the possibility of introducing VTT grading and TT‐GPS score into routine pathologic reports for ccRCC patients to provide further information about risk stratification.

## 2 RESULTS

### 2.1 Evolutionary features during ccRCC progression

**FIGURE 1:**
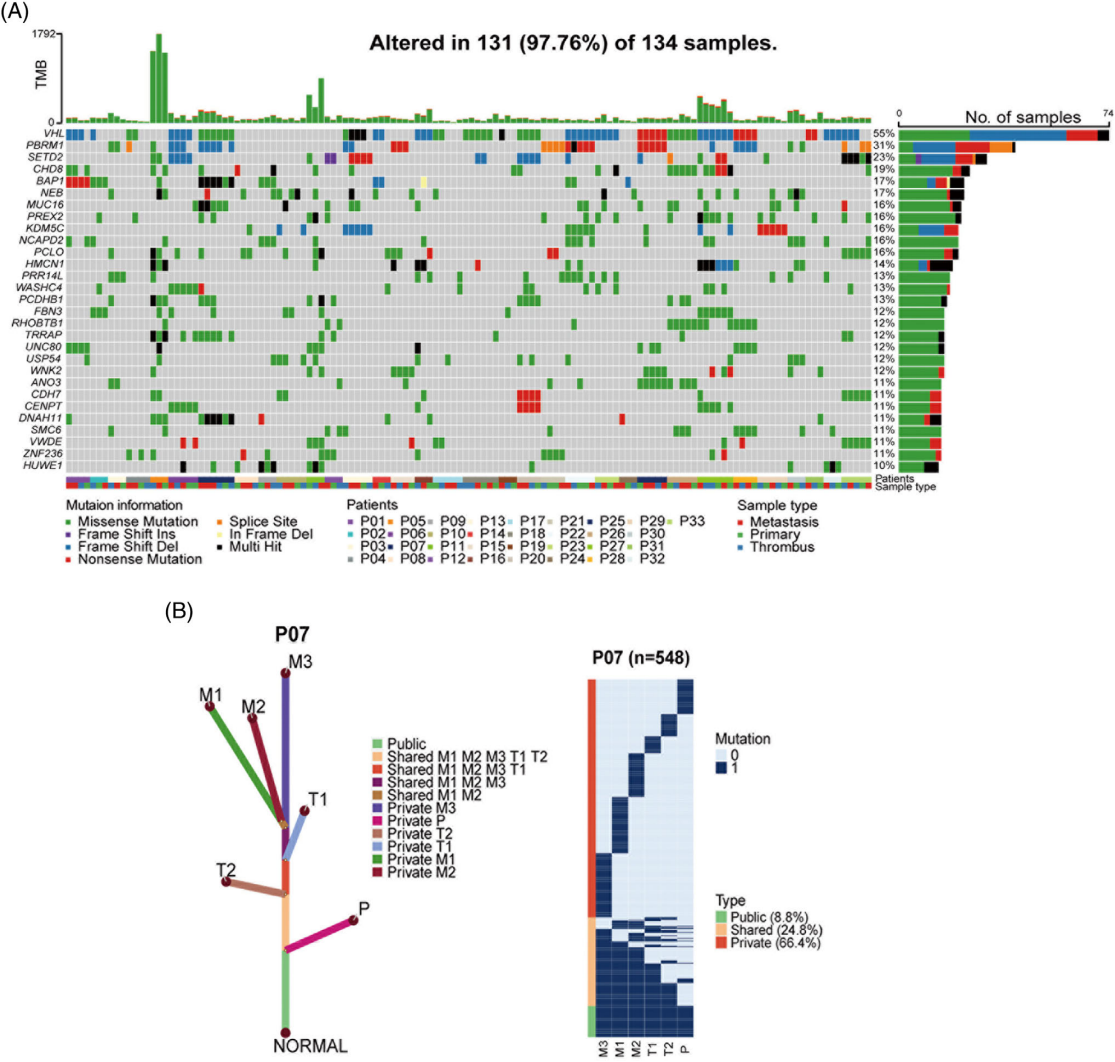
The genomic landscape and phylogenic tree structures. (A) The top panel shows the number of mutations identified in each tumor sample; the middle panel shows mutations of the top 29 driver genes across all samples; the bottom panel shows source of each site. (B) Phylogenic tree structure of evolution relationship among primary (P), thrombus (T) and metastatic (M) of ccRCC patients 07. Heatmap indicates the regional distribution of all mutations in patient 07. The columns next to each heatmap show three categories of mutations and their percentages: public mutations (green); shared mutations (yellow); private mutations (red).

We applied multiregion whole‐exome sequencing (WES) on normal‐tumor‐thrombus‐metastasis quadruples obtained from 33 ccRCC patients (Table S1). A total of 21,645 somatic nonsilent mutations (median 102) were identified in 134 samples (Figure S1), among which VHL (55%), PBRM1 (31%), and SETD2 (23%) were most frequently mutated, consistent with TCGA cohort,^14^ Ma cohort,^5^ and Ding cohort.^15^ Several novel highly mutated genes were identified in our cohort, including CHD8 (19%) and NCAPD2 (16%) (Figure 1A). Intriguingly, phylogenic tree analysis revealed that most metastases (27 out of 33, 81.8%) were seeded from VTT rather than from PTs (representative patient 07; Figures 1B and S2). This result suggested that VTT acted as a reservoir of metastases in the majority of ccRCC patients, inspiring us to explore the role of VTT in the risk assessment of nonmetastatic ccRCC patients.

### 2.2 Patient characteristics

**FIGURE 2:**
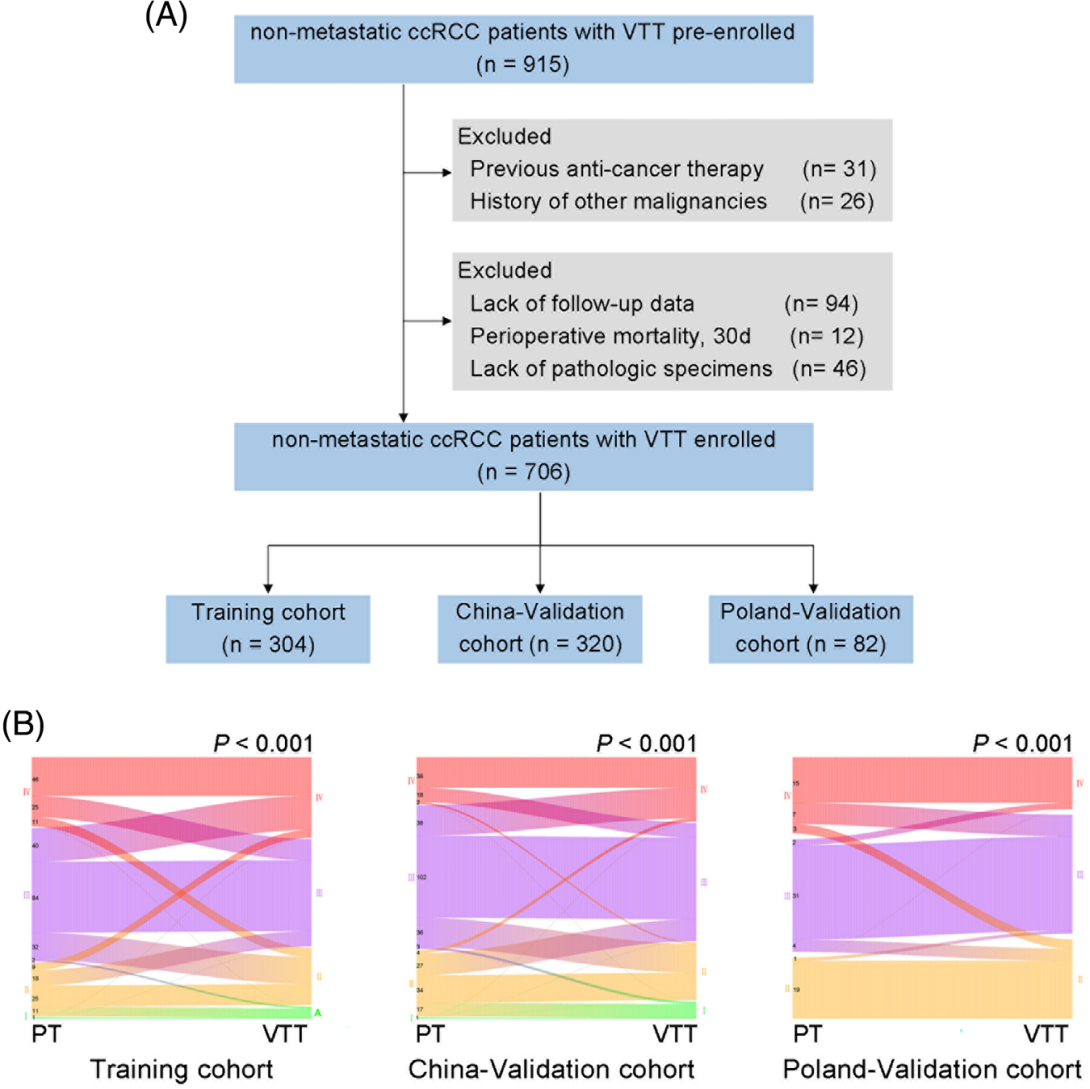
(A) The flow diagram of patient selection. ccRCC, clear cell renal cell carcinoma; VTT, venous tumor thrombus. (B) Sankey diagram illustrating the associations between the PT and VTT grading. Left, China‐Training cohort; Middle, China‐Validation cohort; Right, Poland‐Validation cohort. Significance was assessed by chi‐square test. PT, primary tumor; VTT, venous tumor thrombus.

A total of 915 nonmetastatic ccRCC patients with VTT were pre‐enrolled. Based on the predefined exclusion criteria, the final dataset included 706 patients (Figure 2A). Overall, 446 (63.2%) patients were men, and the median age was 61.5 years (interquartile ranges [IQR], 53−68). The median follow‐up time was 52, 54, and 43 months for Training, China‐Validation, and Poland‐Validation cohorts, respectively. During the follow‐up period, the median OS time was 60, 68, and 68 months for Training, China‐Validation, and Poland‐Validation cohorts, respectively; the median disease‐free survival (DFS) time was 47 and 60 months for Training and China‐Validation cohorts. The clinicopathologic characteristics of the patients were largely balanced among three cohorts (Table S2), except for the major proportion of patients with Mayo 0 in Poland‐Validation cohort.

### 2.3 Pathological grading of VTT signifies distinct prognosis

**FIGURE 3:**
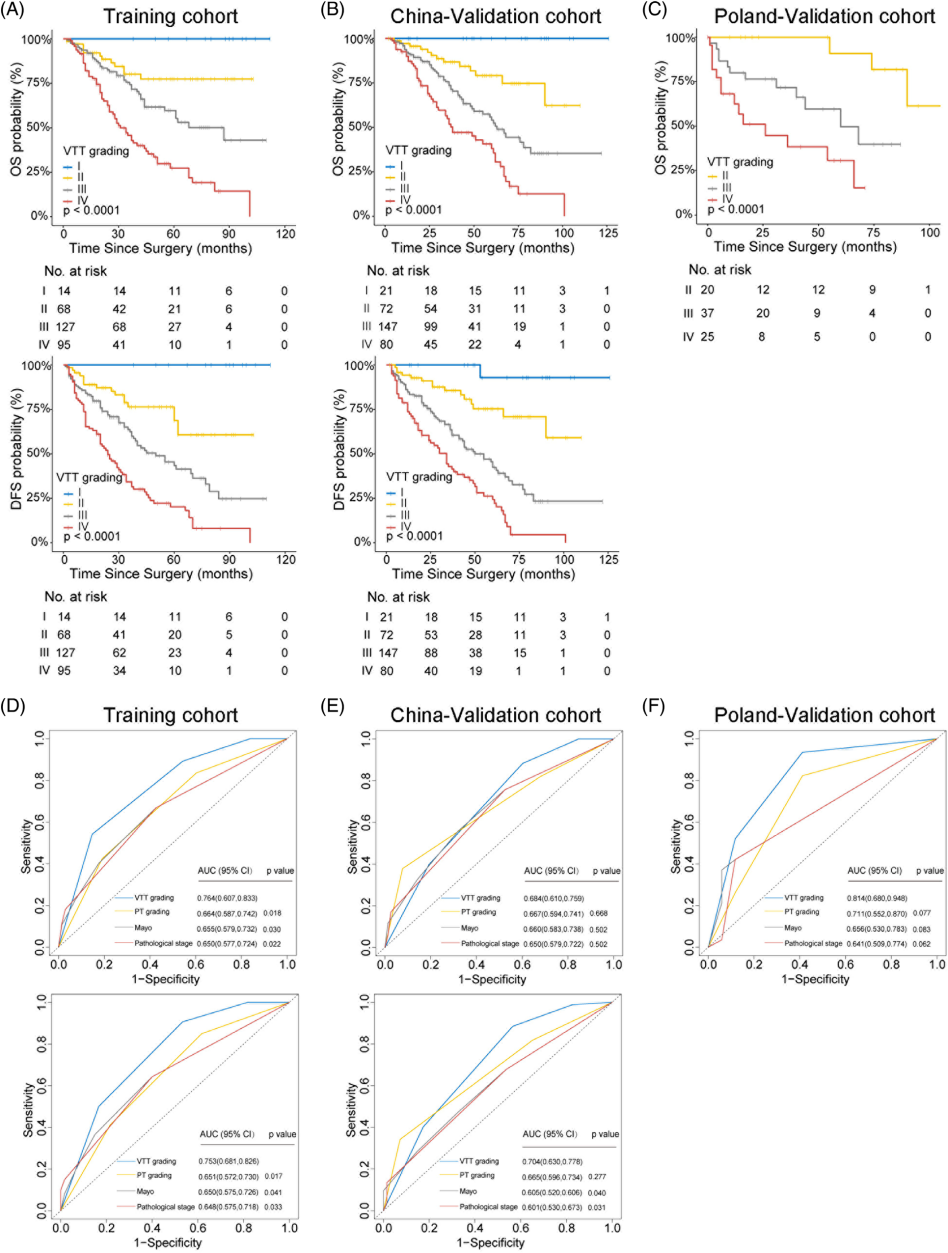
Kaplan–Meier estimates of OS (upper) and DFS (lower) stratified by VTT grading in (A) Training cohort, and (B) China‐Validation cohort, and (C) Poland‐Validation cohort (only OS). ROC curve analysis for comparing the prognostic accuracy of VTT Grading with other variables to predict OS (upper) and DFS (lower) in (D) Training cohort, (E) China‐Validation cohort, and (F) Poland‐Validation cohort. *P* values represented the significant difference between other variables and VTT Grading at 5 years. OS, overall survival; DFS, disease‐free survival; PT, primary tumor; VTT, venous tumor thrombus; AUC; area under the curve; ROC; receiver operating characteristic; CI; confidence interval.

To comprehensively evaluate the potential of VTT in risk assessment, multiple characteristics of VTT were incorporated, including VTT height, consistency and the pathological nuclear grading of VTT (VTT grading), which has not been studied yet. Comparative analysis revealed discrepancies between PT grading and VTT grading in all three cohorts (Figure 2B). Although higher pathological grading of PT and VTT were both significantly correlated with dismal prognosis (Figures 3A–C and Figure S3), only VTT grading remained as an independent predictive factor for OS (Table S3) and DFS (Table S4) after multivariable Cox regression.

Furthermore, VTT grading showed superiority in risk assessment compared with PT grading and other variables by c‐index analysis (OS: 0.663 versus 0.501–0.610, 0.667 versus 0.544–0.651, and 0.719 versus 0.511–0.700 for Training, China‐Validation, and Poland‐Validation cohorts, respectively; DFS: 0.664 versus 0.501–0.606, and 0.672 versus 0.530–0.640 for Training, and China‐Validation cohorts, respectively; Table 1), which was confirmed by the receiver operating characteristic (ROC) analysis (Figures 3D–F; OS: area under the curve [AUC] 0.764 versus 0.650–0.664, 0.684 versus 0.650–0.667, and 0.814 versus 0.641–0.711 for Training, China‐Validation, and Poland‐Validation cohorts, respectively; DFS: AUC 0.753 versus 0.648–0.651, and 0.704 versus 0.601–0.665 for Training, and China‐Validation cohorts, respectively).

**TABLE 1:** Concordance index analysis of the prognostic accuracy of potential variables in indicated cohorts

Variables	OS						DFS			
	Training cohort (*n* = 304)		China‐Validation cohort (*n* = 320)		Poland‐Validation cohort (*n* = 82)		Training cohort (*n* = 304)		China‐Validation cohort (*n* = 320)	
	c‐Index (95% CI)	*p*	c‐Index (95% CI)	*p*	c‐Index (95% CI)	*p*	c‐Index (95% CI)	*p*	c‐Index (95% CI)	*p*
VTT grading	0.663 (0.614–0.713)		0.667 (0.619–0.715)		0.719 (0.638–0.799)		0.664 (0.621–0.707)		0.672 (0.630–0.714)	
PT grading	0.580 (0.531–0.628)	0.002	0.651 (0.606–0.697)	0.518	0.694 (0.618–0.769)	0.474	0.576 (0.532–0.620)	<0.001	0.640 (0.598–0.682)	0.140
Sarcomatoid features in PT	0.593 (0.554–0.632)	0.007	0.624 (0.580–0.667)	0.097	0.617 (0.534–0.700)	0.020	0.563 (0.530–0.595)	<0.001	0.615 (0.579–0.651)	0.012
Sarcomatoid features in VTT	0.547 (0.514–0.580)	<0.001	0.577 (0.539–0.615)	<0.001	0.511 (0.462–0.561)	<0.001	0.543 (0.516–0.571)	<0.001	0.580 (0.547–0.613)	<0.001
Vascular wall invasion	0.548 (0.500–0.597)	0.002	0.544 (0.495–0.593)	0.001	NA		0.547 (0.503–0.590)	<0.001	0.530 (0.486–0.574)	<0.001
Thrombus consistency	0.560 (0.511–0.608)	0.002	0.558 (0.509–0.606)	0.001	NA		0.565 (0.522–0.608)	<0.001	0.584 (0.541–0.627)	0.002
Perirenal fat invasion	0.554 (0.509–0.600)	0.002	0.612 (0.564–0.660)	0.088	0.644 (0.550–0.739)	0.256	0.537 (0.496–0.577)	<0.001	0.594 (0.553–0.636)	0.004
Tumor size	0.501 (0.439–0.562)	<0.001	0.547 (0.492–0.603)	<0.001	0.580 (0.464–0.695)	0.055	0.501 (0.451–0.552)	<0.001	0.530 (0.483–0.578)	<0.001
Pathological T stage	0.604 (0.554–0.654)	0.104	0.607 (0.557–0.656)	0.064	0.688 (0.600–0.776)	0.686	0.606 (0.559–0.652)	0.075	0.567 (0.522–0.612)	0.001
Thrombus level^a^	0.610 (0.557–0.663)	0.149	0.621 (0.567–0.674)	0.172	0.700 (0.604–0.795)	0.791	0.606 (0.558–0.654)	0.073	0.574 (0.526–0.621)	0.001

^a^ According to the Mayo Clinic Classification.^1^

Abbreviations: CI, confidential interval; DFS, disease‐free survival.; NA, not available; OS, overall survival; PT, primary tumor; VTT, venous tumor thrombus.

Additionally, the VTT grading held significance for OS and DFS within the subgroups of patients stratified by clinical and pathological features such as age, adjuvant therapy, tumor size, Mayo clinic classification, PT grading, vascular well invasion, and thrombus consistency (Figures S4–S7). Overall, VTT grading displayed superior accuracy and discriminatory ability in predicting survival risk for nonmetastatic ccRCC patients with VTT.

### 2.4 Development and validation of prognostic model TT‐GPS

**FIGURE 4:**
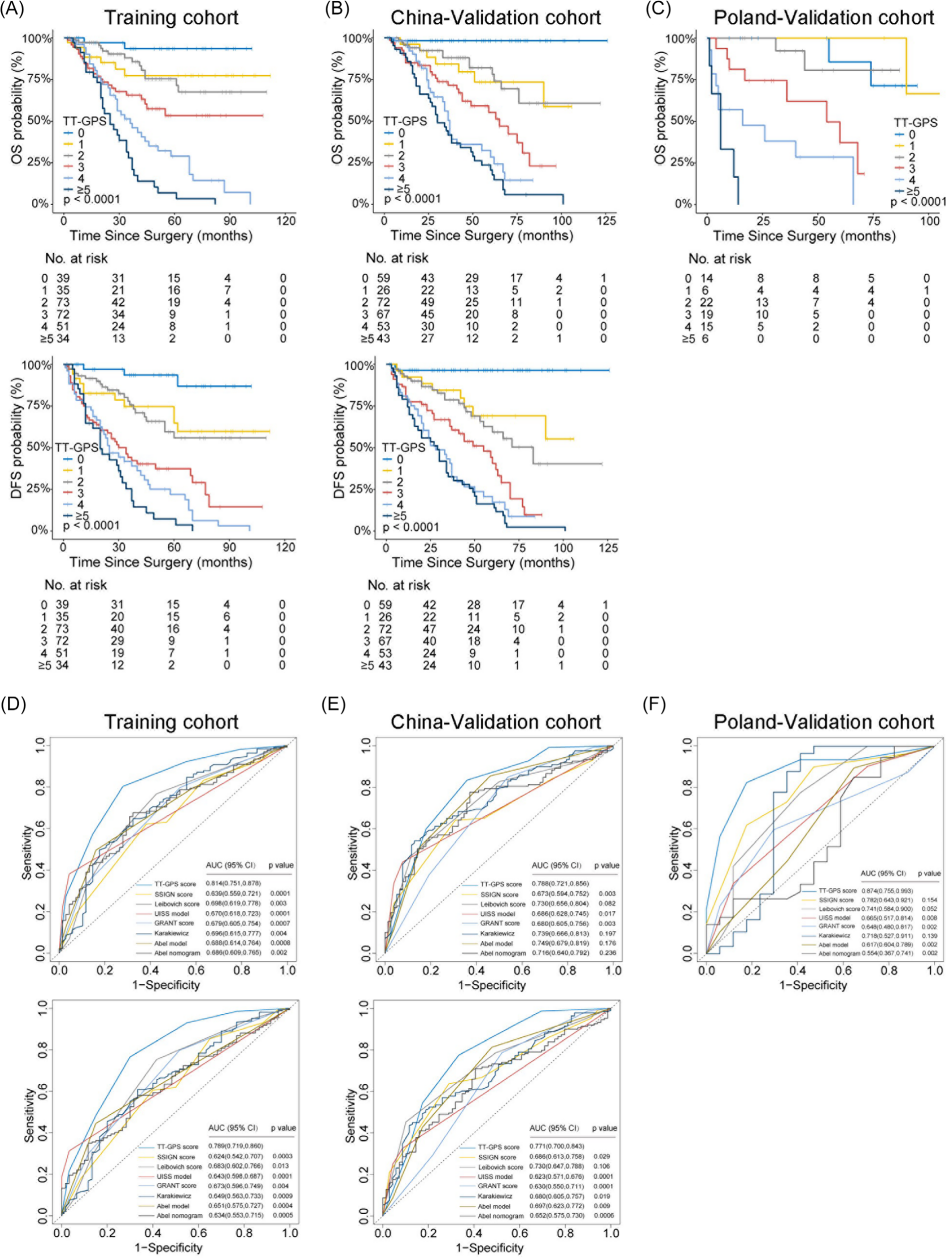
Kaplan–Meier estimates of OS (upper) and DFS (lower) stratified by the TT‐GPS score in (A) Training cohort, (B) China‐Validation cohort, and (C) Poland‐Validation cohort (only OS). ROC curve analysis for comparing the prognostic accuracy of the TT‐GPS score with currently reported prognostic models to predict OS (upper) and DFS (lower) in (D) Training cohort, (E) China‐Validation cohort, and (F) Poland‐Validation cohort. *p* Values represented the significant difference between other variables and VTT grading at 5 years. OS, overall survival; DFS, disease‐free survival; TT‐GPS, VTT height, VTT grading, Perinephric fat invasion, sarcomatoid differentiation in PT. AUC, area under the curve; ROC, receiver operating characteristic; SSIGN, the Mayo Clinic Stage, Size, Grade and Necrosis; UISS, the University of California Los Angeles Integrated Staging System; GRANT, the GRade, Age, Nodes and Tumor; CI, confidence interval.

Based on the above results, a prognostic model incorporating VTT grading may provide more accuracy in risk assessment. Hence, a simple scoring algorithm was developed using the regression coefficients from multivariable Cox analysis in the Training cohort. The coefficient for each independent variable was divided by the coefficient for VTT grading IV, multiplied by 3 and rounded to the nearest integer (Table 2). This model was named as TT‐GPS score, indicating its four variables: VTT height (Mayo Clinic classification), VTT grading, perinephric fat invasion, and sarcomatoid differentiation in PT. The average TT‐GPS score in this study was 2.620 (median 3, range 0–7). Only 32 (4.6%) patients had score of 6 and 7, thus patients with score higher than 5 were combined. Significant differences in OS and DFS among different scores were illustrated by Kaplan–Meier curves in independent cohorts (all *p* < 0.001; Figures 4A–C and Tables S5 and S6).

**TABLE 2:** TT‐GPS score algorithm

Variables	Score
VTT height^a^	
0	0
I	0
II	1
III	1
IV	2
Pathological grading in VTT^b^	
I	0
II	0
III	2
IV	3
Perinephric fat invasion	
No	0
Yes	1
Sarcomatoid differentiation in PT	
No	0
Yes	1

Abbreviations: PT, primary tumor; VTT, venous tumor thrombus.

^a^ According to the Mayo Clinic Classification.^1^

^b^ According to the World Health Organization/International Society of Urological Pathology (WHO/ISUP) grading criterion.^16^

We next compared the TT‐GPS score with previously reported prognostic models, including SSIGN score,^8^ 2003 Leibovich score,^9^ UISS model,^10^ Karakiewicz Nomogram,^3^ and GRANT score^11^ for the general population of RCC patients; Abel model^12^ and Abel nomogram^13^ specific for nonmetastatic RCC patients with VTT. The c‐indices of TT‐GPS score for OS were 0.706 (95% CI, 0.658–0.753) and 0.732 (95% CI, 0.685–0.779) for Training and China‐Validation cohorts, respectively, which were higher than other prognostic models (Table 3). In Poland‐Validation cohort, the TT‐GPS score also outperformed those prognostic models in predicting OS (0.840, 95% CI, 0.791–0.889; Table 3). The superiority of the prognostic accuracy of TT‐GPS score to other prognostic models was confirmed by ROC analysis (Figures 4D–F). Although the predicted probability of TT‐GPS score for 5‐year OS and DFS had concordance with that of the observed probability in all three cohorts, poor calibration of other prognostic models was observed in Poland‐Validation cohort (Figure S8). Moreover, the TT‐GPS score provided consistent positive and larger net benefit across a broad range of risk thresholds compared with other prognostic models by decision curve analysis (DCA) (Figure S9).

**TABLE 3:** Concordance index analysis of the prognostic accuracy of the TT‐GPS score and previously reported prognostic models in indicated cohorts

	OS						DFS			
	Training cohort (*n* = 304)		China‐Validation cohort (*n* = 320)		Poland‐Validation cohort (*n* = 82)		Training cohort (*n* = 304)		China‐Validation cohort (*n* = 320)	
Prognostic model	c‐Index (95% CI)	*p*	c‐Index (95% CI)	*p*	c‐Index (95% CI)	*p*	c‐Index (95% CI)	*p*	c‐Index (95% CI)	*p*
TT‐GPS score	0.706 (0.658–0.753)		0.732 (0.685–0.779)		0.840 (0.791–0.889)		0.691 (0.649–0.732)		0.717 (0.675–0.760)	
SSIGN score	0.584 (0.532–0.637)	<0.001	0.637 (0.587–0.686)	0.001	0.718 (0.611–0.826)	0.028	0.555 (0.506–0.603)	<0.001	0.629 (0.582–0.676)	<0.001
Leibovich score	0.636 (0.586–0.686)	0.005	0.678 (0.632–0.724)	0.016	0.704 (0.610–0.797)	0.003	0.605 (0.559–0.650)	0.002	0.649 (0.603–0.695)	0.001
UISS model	0.640 (0.597–0.683)	0.043	0.644 (0.602–0.686)	0.001	0.732 (0.642–0.822)	0.012	0.622 (0.585–0.659)	0.015	0.595 (0.561–0.629)	<0.001
GRANT score	0.603 (0.551–0.656)	0.002	0.641 (0.587–0.696)	0.002	0.696 (0.613–0.780)	0.001	0.599 (0.553–0.644)	<0.001	0.598 (0.547–0.648)	<0.001
Karakiewicz nomogram	0.630 (0.576–0.684)	0.007	0.684 (0.632–0.736)	0.054	0.672 (0.565–0.779)	0.001	0.603 (0.553–0.654)	0.001	0.641 (0.592–0.690)	<0.001
Abel model	0.629 (0.579–0.680)	0.004	0.672 (0.622–0.722)	0.004	0.714 (0.629–0.800)	0.001	0.603 (0.559–0.648)	<0.001	0.640 (0.594–0.686)	<0.001
Abel nomogram	0.606 (0.546–0.667)	0.004	0.627 (0.567–0.686)	<0.001	0.698 (0.598–0.798)	0.005	0.581 (0.531–0.631)	<0.001	0.591 (0.540–0.643)	<0.001

Abbreviations: CI, confidential interval; DFS, disease‐free survival.; GRANT, the GRade, Age, Nodes and Tumor; OS, overall survival; SSIGN, the Mayo Clinic Stage, Size, Grade and Necrosis; TT‐GPS, VTT height, VTT grading, perinephric fat invasion, sarcomatoid differentiation in PT; UISS, the University of California Los Angeles Integrated Staging System.

**FIGURE 5:**
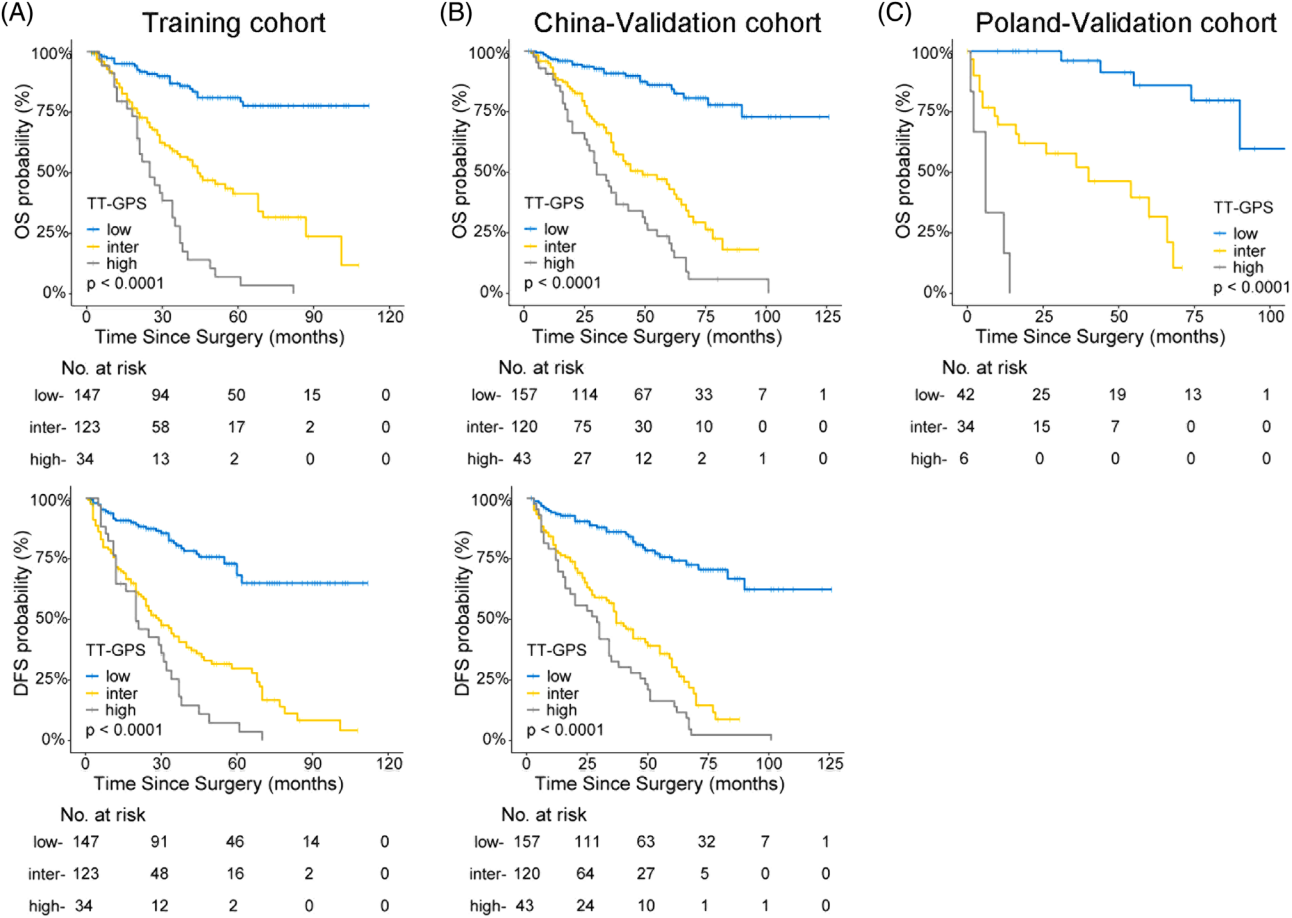
Kaplan–Meier estimates of OS (upper) and DFS (lower) stratified by the TT‐GPS risk classification in (A) Training cohort, (B) China‐Validation cohort, and (C) Poland‐Validation cohort (only OS). OS, overall survival; DFS, disease‐free survival; low, low‐risk group; inter, intermediate‐risk group; high, high‐risk group; TT‐GPS, VTT height, VTT grading, perinephric fat invasion, sarcomatoid differentiation in PT.

For clinical application, three‐tiered risk groups were defined as low (score 0−2), intermediate (score 3 and 4), and high (score ≥ 5) TT‐GPS score by using X‐tile plots. Clinical survival rates were well stratified based on this simple classification (Figure 5 and Table S7 and S8). The patients in the low‐risk group did not reach a median OS and DFS during follow‐up. The median survival of patients in the intermediate‐risk and high‐risk groups was 46 and 29 months for OS and 36 and 21 months for DFS, respectively. Moreover, the TT‐GPS risk classification successfully screened out low‐ and high‐risk patients from those defined as the intermediate‐risk group by previously reported models (Tables S9–S12).

## 3 DISCUSSION

Nonmetastatic ccRCC patients with VTT have variable risk of progression and may possess improved prognosis from accurate risk assessment. By multiregion WES on normal‐tumor‐thrombus‐metastasis quadruples, we offered insights into the notion that VTT served as the main reservoir of distant metastasis.^6^, ^17^ However, the clinicopathological features and prognostic potential of VTT have not been systematically evaluated.

To date, pathological reports only describe whether there are tumor cells within VTT in routine clinical practice. Our data comprehensively evaluated multiple characteristics of VTT and showed for the first time that VTT grading represented an unheeded prognostic predictor and outperformed conventional indicators, including PT grading, VTT height and so on. There was no significant difference of tumor cell percentages in VTT specimens among four‐tired grading, and no significant difference of Ki67 proliferative index between PT and VTT specimens, indicating that the predictive value of VTT grading is independent of tumor proliferation activity.

Nonmetastatic ccRCC patients with VTT, belonging to locally advanced RCC, are ideal candidates for adjuvant therapy. However, accepting adjuvant therapy had no significance in improving the survival of these patients in our study (*p* > 0.05; Table S2). The reason may be that there is heterogeneity of progression and survival risk among patients.^18^ The high‐risk subgroup identified by TT‐GPS score might be candidates for further aggressive treatment, such as immunotherapy‐based therapy. In addition, the TT‐GPS score is simple to calculate and all variables are routinely available clinical and pathological features.

Our study is not devoid of limitations. The retrospective nature introduces the risk of measurement and ascertainment biases. Additional cohorts from non‐Asian patients are needed to confirm our findings, considering the relatively small sample size of external validation. Since the TT‐GPS score is developed and validated only for clear cell histology, further work is needed to expand the score on papillary histology.

In conclusion, we showed that VTT grading represents an unheeded prognostic predictor. Moreover, the TT‐GPS score we developed and validated allows for accurate risk positioning, outperforms previously reported models. Further validation of TT‐GPS score is currently underway in different ethnic populations to confirm its value. Our study highlights the possibility of introducing VTT grading and TT‐GPS score into routine pathological reports to provide further information for risk stratification.

## 4 MATERIALS AND METHODS

The study was approved by the institutional review board of initiating center Jinling Hospital (ID Number: 2021NZKY‐004‐01). Written informed consent was obtained from all patients. This study was conducted adhering to guidelines of Declaration of Helsinki for biomedical research and the Transparent Reporting of a multivariable prediction model for Individual Prognosis Or Diagnosis (TRIPOD)^19^ (Table S13).

### 4.1 DNA isolation and WES

We collected 164 samples for WES from 33 ccRCC patients with matched tumor‐thrombus‐metastasis quadrupled samples from the Eastern China Renal Cancer Collaborative Group, including 30 normal tissues, 40 PT samples, 41 VTT samples, and 53 metastasis samples. To avoid the impact of intratumor heterogeneity, multiregions of the same specimens were sampled by a margin of at least 0.5 cm. Every histologic section was independently reviewed by three pathologists to ensure the quality of the specimens. Genomic DNA from formalin‐fixed paraffin‐embedded (FFPE) samples was extracted using the QIAamp DNA FFPE Tissue Kit (Qiagen). A total of 1 μg DNA were extracted from each sample and sheared into fragment by Covaris (M22). The Equalbit® dsDNA HS Assay Kit was utilized to measure the library concentration; then the Agilent 4200 TapeStation System was utilized to detect the distribution of library fragments, and finally the KAPA Library Quant kit (illumina) universal qPCR Mix was adopted to accurately determine the molar concentration of the library. The gDNA libraries were subjected to high‐throughput sequencing with 150‐bp pair‐end reads on the NovaSeq 6000 Sequencing System (Illumina, San Diego, CA). The average sequencing depth was 150× for tumors and 100× for normal tissues. Details of raw data processing, alignment and somatic mutation calling for genomes were performed with standard protocols, which were described previously.^20^, ^21^

### 4.2 Patient cohorts

We only collected ccRCC patients based on the vast majority proportion of this subtype with VTT.^22^ Patients with previous anticancer therapy, history of other malignancies (biasing the outcome of prognosis), lack of follow‐up data, lack of pathologic specimens, and perioperative mortality (first month after surgery) were excluded from the analysis. Thus, the final evaluable dataset enrolled 706 consecutively nonmetastatic ccRCC patients who underwent radical nephrectomy and thrombectomy, including 304 in the Training cohort from the Eastern China Renal Cancer Collaborative Group, 320 in the China‐Validation cohort and 82 in the Poland‐Validation cohort.

Follow‐up was performed according to the institutional protocols, which was executed postoperatively at least every 3−6 months for the first 5 years and annually thereafter. The primary outcome was OS, defined as the interval from the date of radical surgery to the date of death from any cause, or the last follow‐up period. The secondary outcome was DFS, defined as the interval from the date of radical surgery to the date of radiological evidence of tumor progression, death from any cause, or the last follow‐up. All follow‐ups were concluded in July 2022.

### 4.3 Clinical variables

Standard preoperative assessments were similar among each institution and included laboratory and radiographic evaluation (computed tomography (CT) or magnetic resonance imaging scans of chest, abdomen, and pelvis). Positron emission tomography/CT was utilized for suspected metastasis. Clinical variables were evaluated for each patient including age at surgery, gender, body mass index, presence of pain or hematuria, hypertension, diabetes, tumor laterality, and thrombus height. The thrombus height was defined according to Mayo Clinic Classification.^1^ Preoperative laboratory variables included serum creatinine, albumin, hemoglobin, and neutrophil to lymphocyte ratio. Operative risk factors included surgical approach (open or laparoscopic), surgical time, and blood transfusion. Following the European Association of Urology Guidelines,^2^ decisions about the surgical approach were made by the primary surgeon based on individual patient/tumor characteristics.

### 4.4 Pathological variables

Pathological variables including the World Health Organization/International Society of Urological Pathology (WHO/ISUP) grading,^16^ perinephric fat invasion, presence of tumor necrosis, presence of sarcomatoid differentiation, and rhabdoid differentiation were evaluated in both PT and VTT specimens. VTT grading was based on the highest grading present on any slide, even if focal, as the same as PT grading.^16^ Specifically, ISUP grading I were defined as having inconspicuous or absent nucleoli at ×400 magnification; for ISUP grading II, nucleoli should be distinctly visible at ×400, but inconspicuous or invisible at ×100 magnification; and for ISUP grading III, nucleoli should be distinctly visible at ×100 magnification. ISUP grading IV was defined by the presence of pronounced nuclear pleomorphism, tumor giant cells, and/or rhabdoid and/or sarcomatoid differentiation.^23^ The thrombus consistency^24^ and vascular wall invasion^25^ were also determined in VTT specimens. The TNM stage was determined according to the 8th edition American Joint Committee on Cancer (AJCC) classification.^26^ All pathological specimens were centrally reviewed by two genitourinary pathologists (Hui Chen and Yao Fu, with 12 and 8 years of experience in uropathology, respectively) blinded to clinical information. When there was a different opinion, a third pathologist (Qiu Rao with 22 years of experience in uropathology) re‐evaluated the slice and finally reached a consensus.

### 4.5 Sample size calculation

According to the previous studies about PT grading in nonmetastatic ccRCC patients with VTT,^12^, ^13^ we assumed that the proportion of VTT grading I–II was about 30% and the hazard ratio (HR) of death was 3 for VTT grading III–IV versus I–II. The overall death rate was 40% during follow‐up in our study. Every cohort needed at least 78 patients to obtain statistically significant HR with a two‐sided test at a significance level of 0.05 and a power of 0.8. Moreover, the 5‐year OS rates were assumed to be 50% in the high‐risk group and 80% in the low‐risk group according to our prognostic model. Every cohort needed at least 62 patients to detect the difference with a two‐sided log‐rank test at a significance level of 0.05 and a power of 0.8 in an average 5‐year follow‐up. Therefore, a minimum sample size of 78 patients was required for each cohort. The procedure “Cox Regression” and “Logrank Tests” in PASS 15 was used to calculate the sample size.

### 4.6 Statistical analysis

Categorical variables, normally distributed continuous variables, and non‐normally distributed continuous variables were reported as numbers and percentages, means and standard deviations, medians and IQR, respectively. The ANOVA tests were utilized to test differences between multiple groups for continuous variables assuming normal distribution and homogeneity of variance. If not, Kruskal–Wallis tests would be applied. Chi‐square test and Cochran–Mantel–Haenszel Chi‐square test were used to test differences between categorical variables and ordinal variables. The Kaplan–Meier method with log‐rank test was used for survival analysis and comparisons. Univariable and multivariable Cox regression analyses were performed to identify independent predictors associated with survival outcomes. Factors significant on univariable cox regression were evaluated by stepwise cox regression with 0.05 for entry and 0.1 for staying. The missing rate of the variables included in our data was relatively low, mostly between 0 and 3% and all variables except for pathological characteristics were integral in these cohorts. Therefore, as primary analysis, we conducted a complete‐case analysis using the participants with complete data. Pathological stage was excluded in the multivariable analysis because of collinearity with Mayo Clinic classification, and Spearman correlation coefficients were 0.95, 0.93 and 0.99 in three cohorts. We evaluated the discrimination or prognostic accuracy of prognostic indicators or models using Harrell's concordance index (c‐index), which is appropriate for censored data.^27^ We also used time‐dependent ROC analysis and AUC to measure prognostic accuracy. The discrimination of models was compared by c‐index^28^ and AUC.^29^ Calibration plots were generated to assess how closely the predicted outcomes approximated the actual outcomes.^30^ Clinical usefulness of the prediction model was assessed by DCA by quantifying the net benefits at different threshold probabilities.^31^ All statistical tests were two‐sided with a significance level set at 0.05. All statistical analyses were performed using SAS software version 9.4 (SAS Institute Inc., Cary, NC, USA), except c‐index, ROC, calibration plots, and DCA using R 4.0.4. (R Project for Statistical Computing, Vienna, Austria).

## AUTHOR CONTRIBUTION

L. Q. and L. H. W. were the overall principal investigators who conceived the study and obtained financial support. W. Q. Z., J. P. G., L. H. W., X. F., B. K. S., S. G. W., J. H. Z., W. X., M. C., H. Q. G., X. B. Z., W. J. Q., H. F. W., D. X., N. X., C. Z. L., G. X. W., Y. X. L., H. W. Z., Z. Y. L., Ł. Z., and Z. W. contributed to provision of study material or patients. S. L. G., N. W. Y., Y. L. Z., Z. J. W., Y. Z., T. L. Z., Y .F .G., H. M., H. W. H., X. M. Y., C. P. T., D. F., R. C., X. Z., G. Y. Z., C. W. J., C. Z., Y. M. L., C. C. F., T. L. J., S. H. C., S. T., C. Z., L. W., and W. Z. contributed to acquisition of data, including patient information and slice collection. H. C., Y. F., and Q. R. contributed to the pathological review of slides. L. Q., Q. C., A. M. J., M. X. H., B. H., X. F., M. K., J. L. G., D. S., and C. C. contributed to statistical analysis and interpretation of the results. L. Q., Y. M. L., and C. C. contributed to writing, review, or revision of the manuscript. All authors have read and approved the final manuscript.

## CONFLICT OF INTEREST STATEMENT

All authors declare no competing interests.

## ETHICS STATEMENT

The study was approved by the institutional review board of initiating center Jinling Hospital (ID Number: 2021NZKY‐004‐01). Written informed consent was obtained from all patients. This study was conducted adhering to guidelines of Declaration of Helsinki for biomedical research and the Transparent Reporting of a multivariable prediction model for Individual Prognosis Or Diagnosis (TRIPOD).

## Supporting information

Supporting Information

## Data Availability

The datasets generated during and analyzed during the current study are available by the corresponding author, upon reasonable request. The WES data have been deposited (PRJCA014721) in the Genome Sequence Archive of Human in the BIG Data Center which are publicly accessible at https://bigd.big. ac.cn/gsa‐human.
