# Supplementary material for: Development and validation of a prognostic model incorporating tumor thrombus grading for nonmetastatic clear cell renal cell carcinoma with tumor thrombus: A multicohort study
Source: MedComm (2020). 2023 Jul 20;4(4):e300. doi: 10.1002/mco2.300 (PMC10357251; doi:10.1002/mco2.300)
Supplement: Supplementary file 1 — Supporting Information [file MCO2-4-e300-s001.pdf]

Supplemental information

Development and Validation of a Prognostic Model Incorporating Tumor Thrombus Grading for Non-metastatic Clear Cell Renal Cell Carcinoma with Tumor Thrombus: A Multi-cohort Study

Le Qu; Hui Chen; Qi Chen; Silun Ge; Aimin Jiang; Nengwang Yu; Yulin Zhou; Michał Kunc; Ye Zhou; Xiang Feng; Wei Zhai; Zhenjie Wu; Miaoxia He; Yaoming Li; Rui Chen; Bo Han; Xing Zeng; Yao Fu; Changwei Ji; Xiang Fan; Guangyuan Zhang; Cheng Zhao; Taile Jing; Chenchen Feng; Hongwei Zhao; Di Sun; Liang Wang; Sheng Tai; Cheng Zhang; Shaohao Chen; Yixun Liu; Haifeng Wang; Jinli Gao; Yufeng Gu; He Miao; Tangliang Zhao; Xiaoming Yi; Chaopeng Tang; Dian Fu; Haowei He; Qiu Rao; Wenquan Zhou; Ning Xu; Gongxian Wang; Chaozhao Liang; Zhiyu Liu; Dan Xia; Xiongbing Zu; Ming Chen; Hongqian Guo; Weijun Qin; Zhe Wang; Wei Xue; Benkang Shi; Shaogang Wang; Junhua Zheng; Cheng Chen; Łukasz Zapała; Jingping Ge; Linhui Wang

Supplementary Methods

Raw WES data processing and visualization

Sequence reads were aligned to human genome hg38 using BWA, followed with the GATK best practice workflow to remove duplications and BQSR. For somatic mutations (SNVs and INDELs), we took the intersection of variant calls from MuTect2 and Strelka2 software (The samples that lack normal controls used a tumor-only mode of Mutect2). The visualization of mutation landscape and phylogenic tree analysis were performed with the use of R software (Version 4.2), and R packages maftools[1] and meskit.[2]

Participating centers

The Training cohort included 304 patients consecutively collected between 2012 and 2021 from the Eastern China Renal Cancer Collaborative Group in China (Training cohort): Jinling Hospital; Changhai Hospital; Qilu Hospital, Renji Hospital, Drum Tower Hospital, Zhongda Hospital, The First Affiliated Hospital of Zhejiang University, The First Affiliated Hospital of Fujian Medical University, The First Affiliated Hospital of Anhui Medical University, and The First Affiliated Hospital of Nanchang University; while the China-Validation cohort included 320 patients consecutively collected between 2012 and 2021 from Tongji Hospital, Xiangya Hospital, Huashan Hospital, Yuhuangding Hospital, Southwest Hospital, Xijing Hospital, Daping Hospital, Shanghai East Hospital, Changzheng Hospital, The First Affiliated Hospital of University of Science and Technology of China, The First Affiliated Hospital of Shandong First Medical University, The First Affiliated Hospital of Guangxi Medical University, The Second Affiliated Hospital of Dalian Medical University. The Poland-Validation cohort comprised 82 patients consecutively collected between 2012 and 2018 from the Medical University of Gdańsk and the Medical University of Warsaw in Poland.[3]

Supplementary References

1. Mayakonda A, Lin D-C, Assenov Y, Plass C, Koeffler HP. Maftools: efficient and comprehensive analysis of somatic variants in cancer. *Genome research*. 2018;28(11):1747-1756.
2. Liu M, Chen J, Wang X, et al. MesKit: a tool kit for dissecting cancer evolution of multi-region tumor biopsies through somatic alterations. *Gigascience*. 2021;10(5):giab036.
3. Zapała Ł, Sharma S, Kunc M, et al. Analysis of Clinicopathological Factors Influencing Survival in Patients with Renal Cell Carcinoma and Venous Tumor Thrombus. *Journal of Clinical Medicine*. 2021;10(17):3852.

Figure S1. (A) Mutation summary of 33 ccRCC patients. (B) Number of mutations of each sample from 33 ccRCC patients.

A

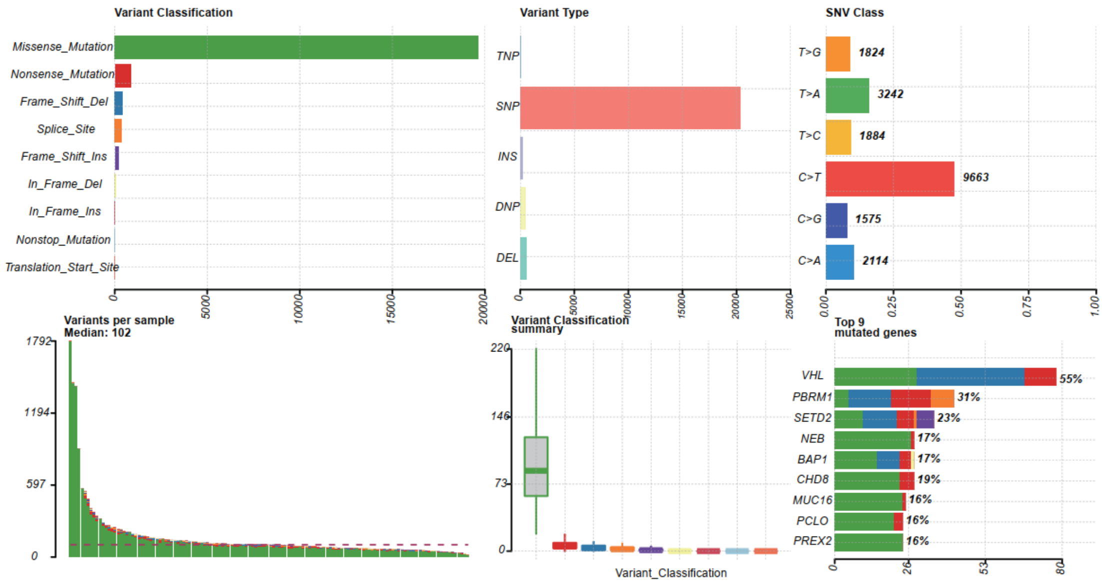

B

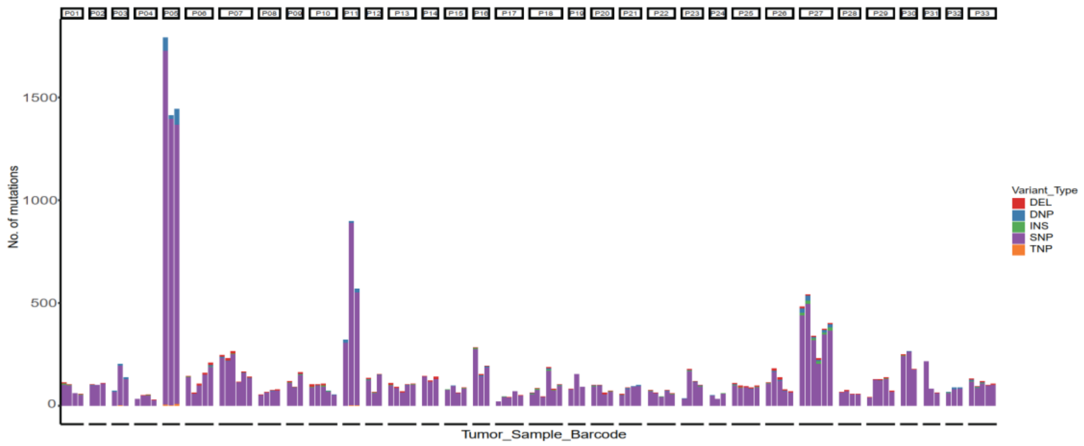

Figure S2. Phylogenic tree structures of evolution relationship among primary (P), thrombus (T) and metastatic (M) of the remained 31 ccRCC patients.

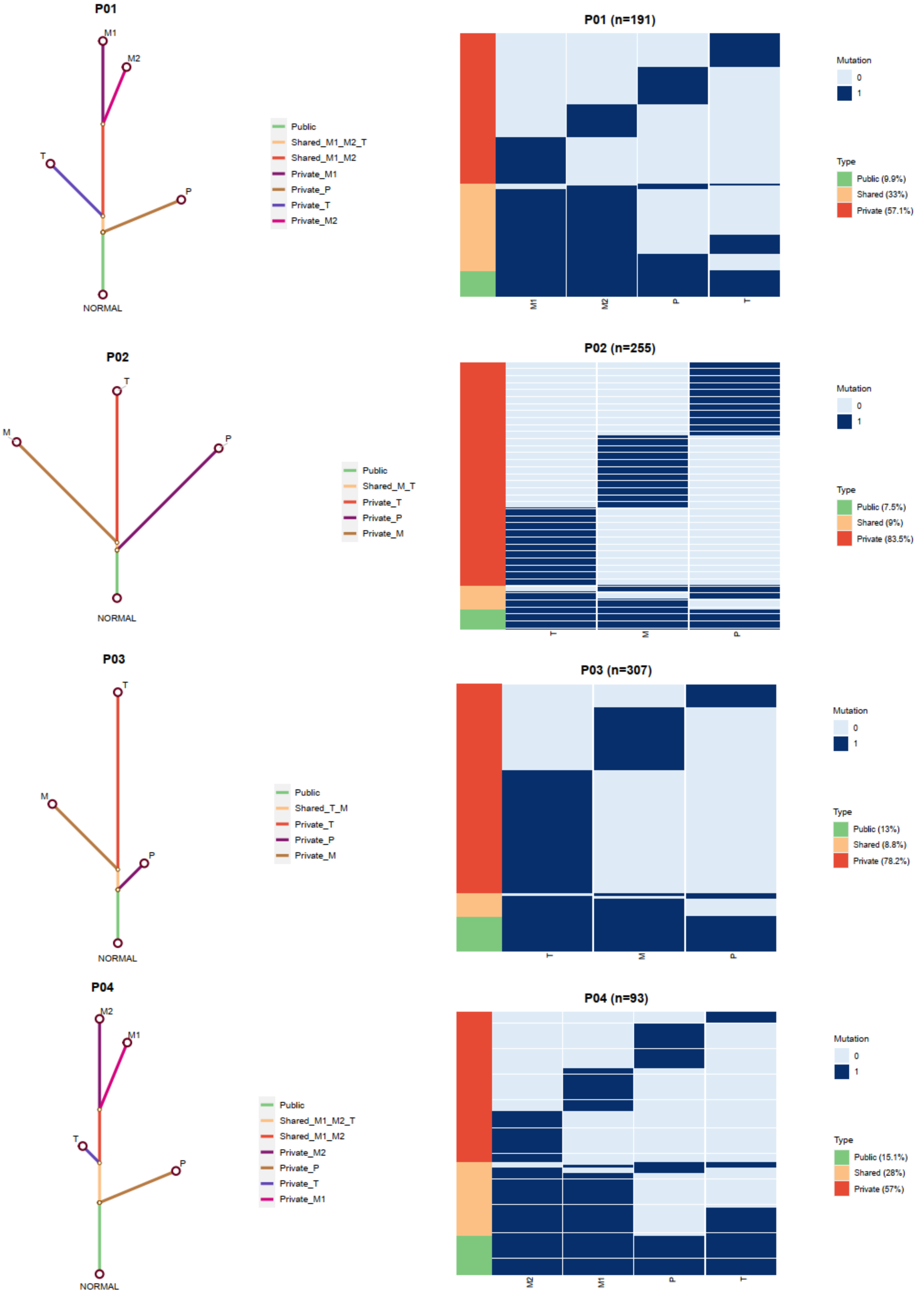

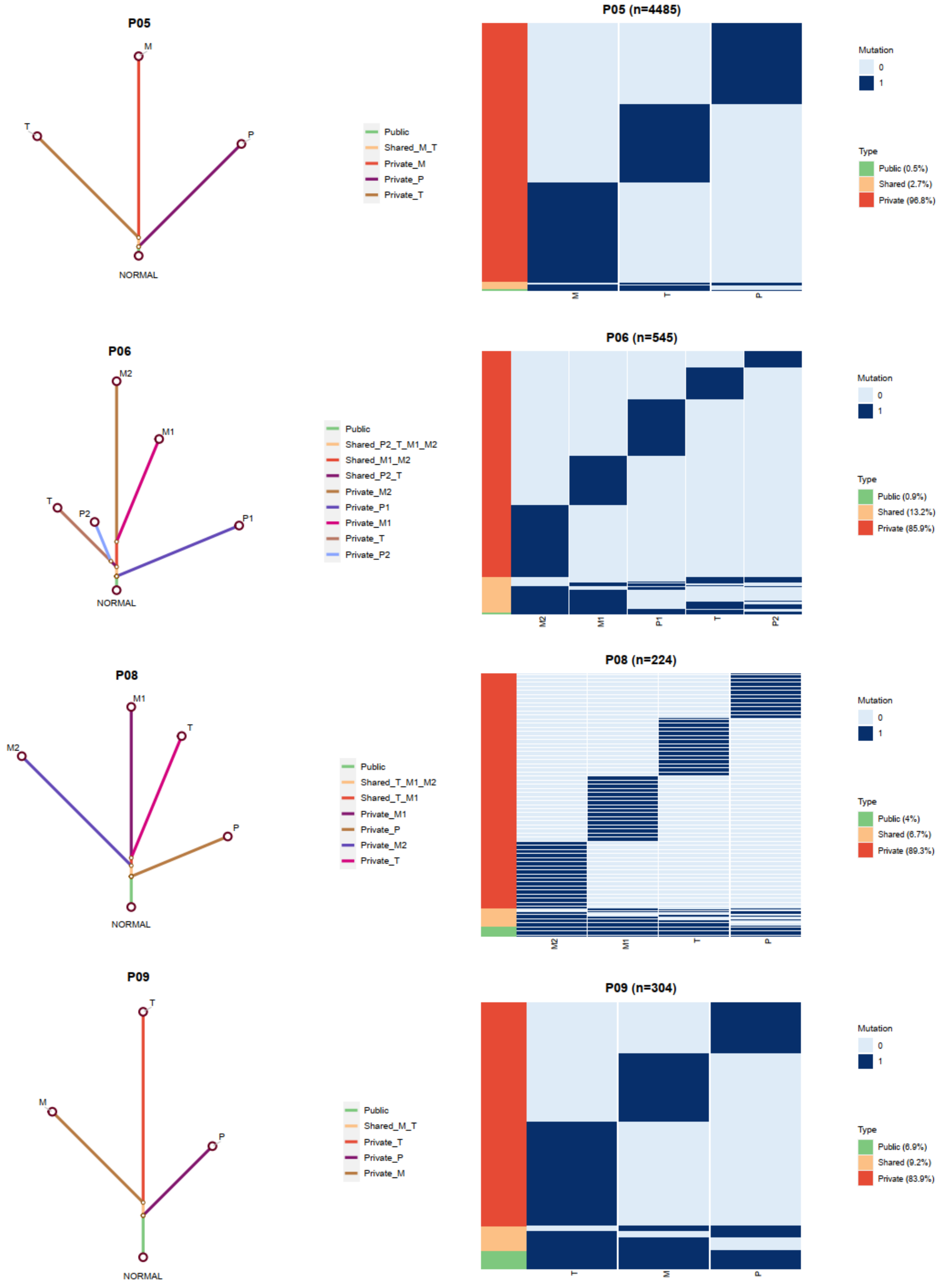
P05
M
T
P
NORMAL
Public
Shared_M_T
Private_M
Private_P
Private_T
P05 (n=4485)
M
T
P
Mutation
0
1
Type
Public (0.5%)
Shared (2.7%)
Private (96.8%)
P06
M2
M1
T
P2
P1
NORMAL
Public
Shared_P2_T_M1_M2
Shared_M1_M2
Shared_P2_T
Private_M2
Private_P1
Private_M1
Private_T
Private_P2
P06 (n=545)
M2
M1
P1
T
P2
Mutation
0
1
Type
Public (0.9%)
Shared (13.2%)
Private (85.9%)
P08
M1
T
M2
P
NORMAL
Public
Shared_T_M1_M2
Shared_T_M1
Private_M1
Private_P
Private_M2
Private_T
P08 (n=224)
M2
M1
T
P
Mutation
0
1
Type
Public (4%)
Shared (6.7%)
Private (89.3%)
P09
T
M
P
NORMAL
Public
Shared_M_T
Private_T
Private_P
Private_M
P09 (n=304)
T
M
P
Mutation
0
1
Type
Public (6.9%)
Shared (9.2%)
Private (83.9%)

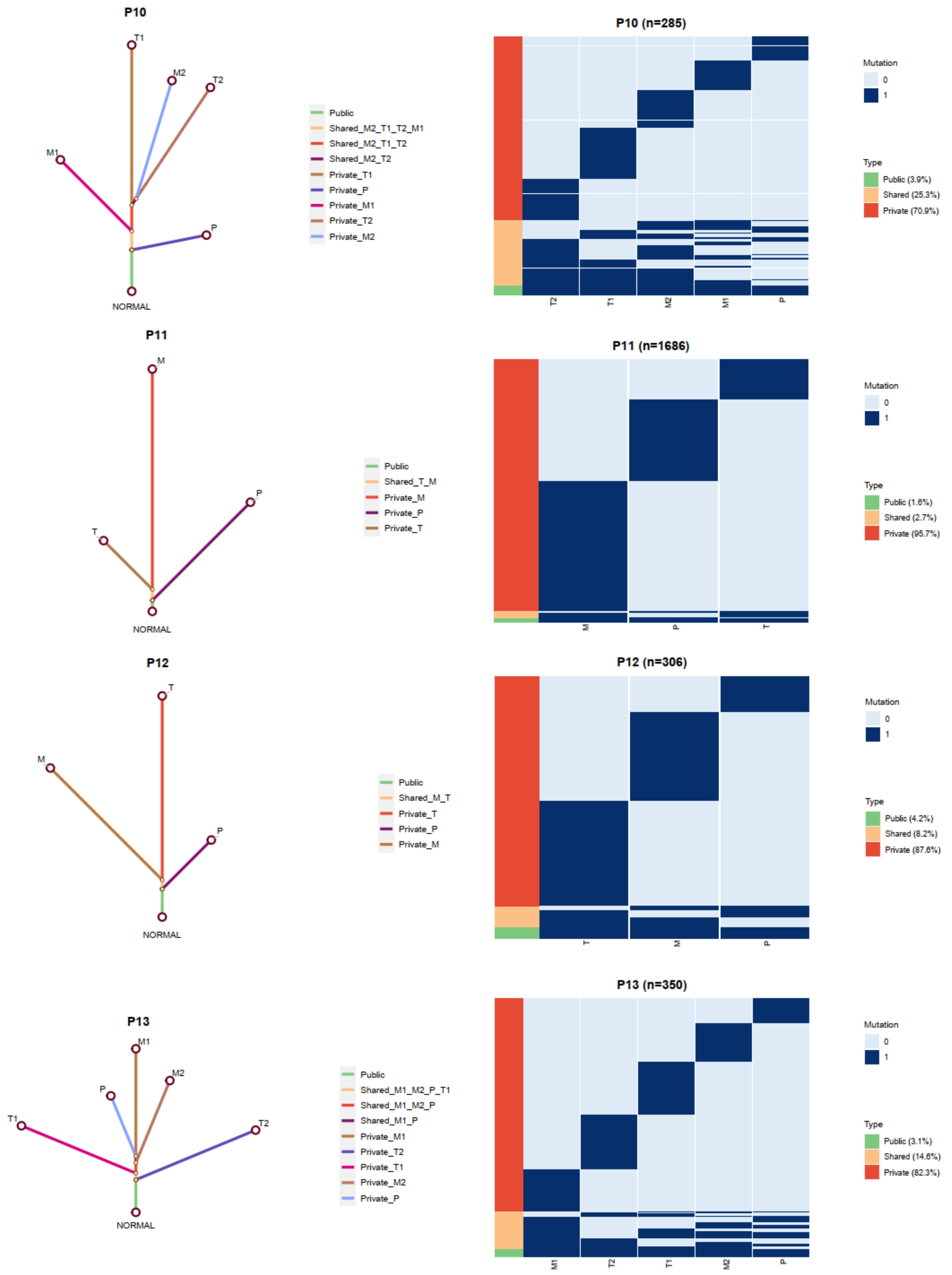
P10
T1
M2
T2
M1
P
NORMAL
Public
Shared_M2_T1_T2_M1
Shared_M2_T1_T2
Shared_M2_T2
Private_T1
Private_P
Private_M1
Private_T2
Private_M2
P10 (n=285)
T2
T1
M2
M1
P
Mutation
0
1
Type
Public (3.9%)
Shared (25.3%)
Private (70.9%)
P11
M
P
T
NORMAL
Public
Shared_T_M
Private_M
Private_P
Private_T
P11 (n=1686)
M
P
T
Mutation
0
1
Type
Public (1.6%)
Shared (2.7%)
Private (95.7%)
P12
T
M
P
NORMAL
Public
Shared_M_T
Private_T
Private_P
Private_M
P12 (n=306)
T
M
P
Mutation
0
1
Type
Public (4.2%)
Shared (8.2%)
Private (87.6%)
P13
M1
M2
P
T1
T2
NORMAL
Public
Shared_M1_M2_P_T1
Shared_M1_M2_P
Shared_M1_P
Private_M1
Private_T2
Private_T1
Private_M2
Private_P
P13 (n=350)
M1
T2
T1
M2
P
Mutation
0
1
Type
Public (3.1%)
Shared (14.6%)
Private (82.3%)

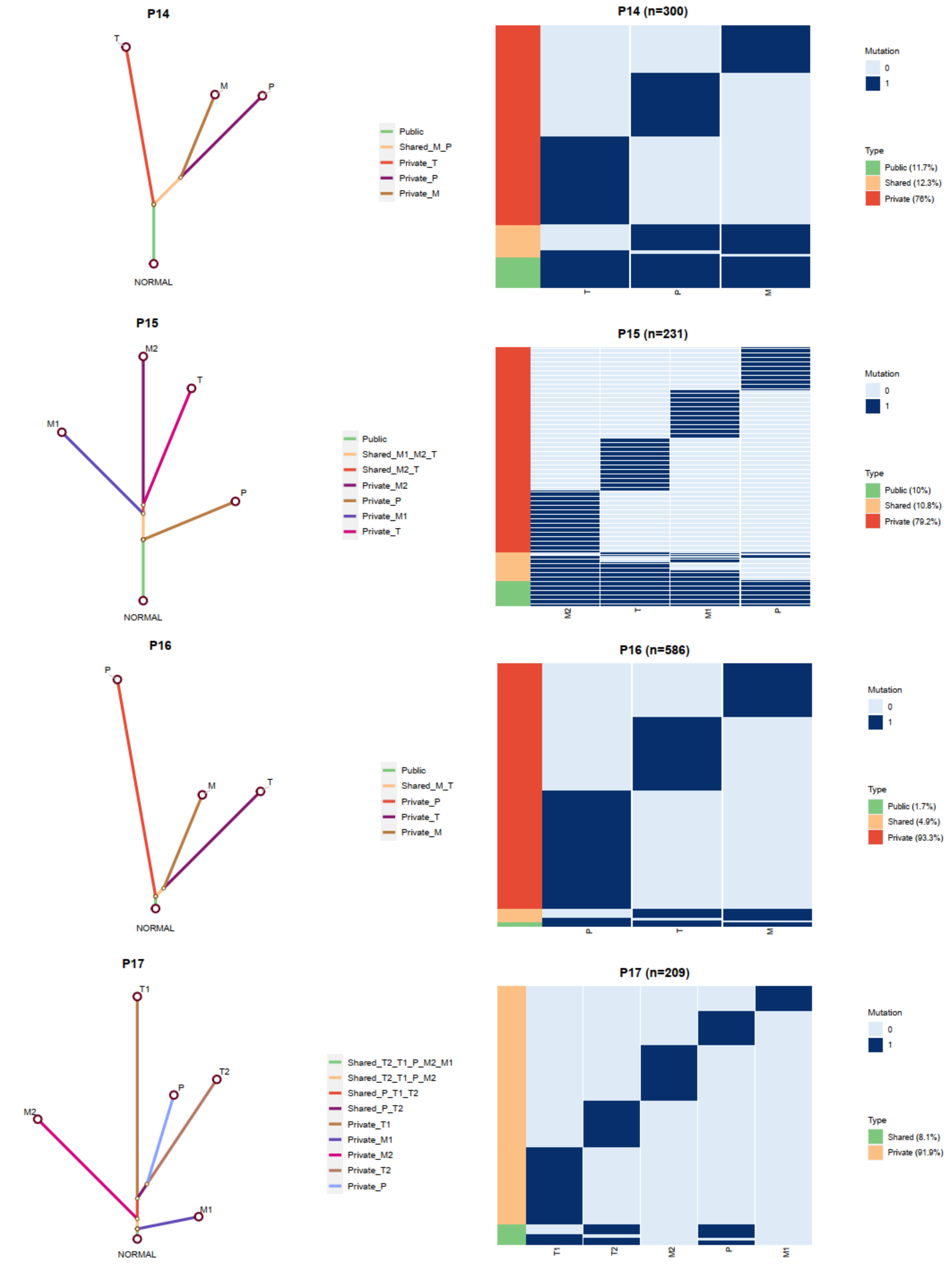
P14
T
M
P
NORMAL
Public
Shared_M_P
Private_T
Private_P
Private_M
P14 (n=300)
Mutation
0
1
Type
Public (11.7%)
Shared (12.3%)
Private (76%)
T
P
M
P15
M2
T
M1
P
NORMAL
Public
Shared_M1_M2_T
Shared_M2_T
Private_M2
Private_P
Private_M1
Private_T
P15 (n=231)
Mutation
0
1
Type
Public (10%)
Shared (10.8%)
Private (79.2%)
M2
T
M1
P
P16
P
M
T
NORMAL
Public
Shared_M_T
Private_P
Private_T
Private_M
P16 (n=586)
Mutation
0
1
Type
Public (1.7%)
Shared (4.9%)
Private (93.3%)
P
T
M
P17
T1
P
T2
M2
M1
NORMAL
Shared_T2_T1_P_M2_M1
Shared_T2_T1_P_M2
Shared_P_T1_T2
Shared_P_T2
Private_T1
Private_M1
Private_M2
Private_T2
Private_P
P17 (n=209)
Mutation
0
1
Type
Shared (8.1%)
Private (91.9%)
T1
T2
M2
P
M1

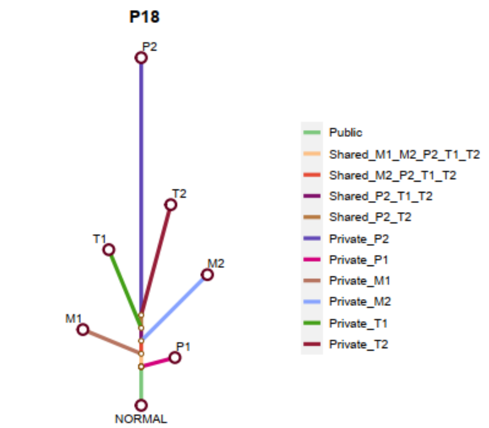
P18
P2
T2
T1
M2
M1
P1
NORMAL
Public
Shared_M1_M2_P2_T1_T2
Shared_M2_P2_T1_T2
Shared_P2_T1_T2
Shared_P2_T2
Private_P2
Private_P1
Private_M1
Private_M2
Private_T1
Private_T2

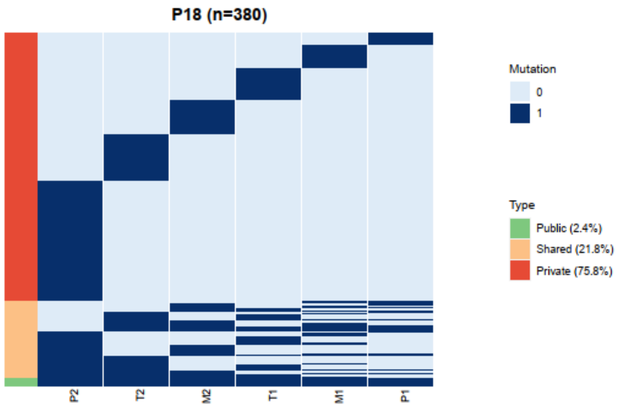
P18 (n=380)
P2
T2
M2
T1
M1
P1
Mutation
0
1
Type
Public (2.4%)
Shared (21.8%)
Private (75.8%)

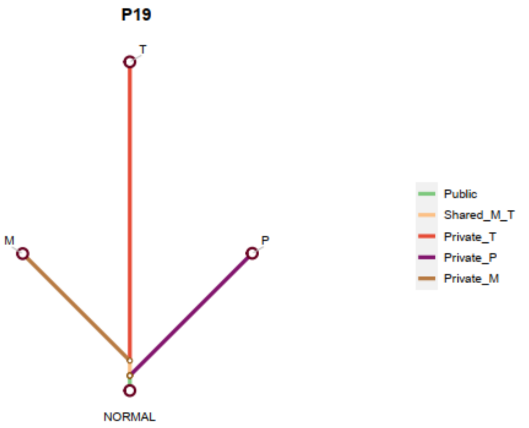
P19
T
M
P
NORMAL
Public
Shared_M_T
Private_T
Private_P
Private_M

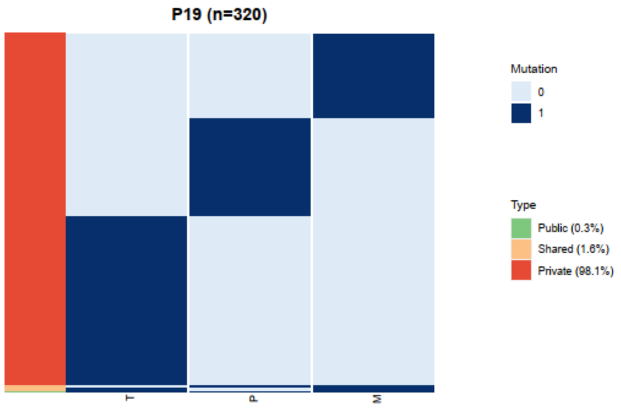
P19 (n=320)
T
P
M
Mutation
0
1
Type
Public (0.3%)
Shared (1.6%)
Private (98.1%)

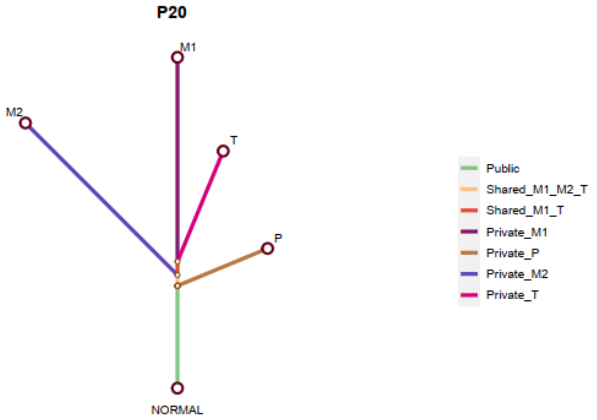
P20
M1
M2
T
P
NORMAL
Public
Shared_M1_M2_T
Shared_M1_T
Private_M1
Private_P
Private_M2
Private_T

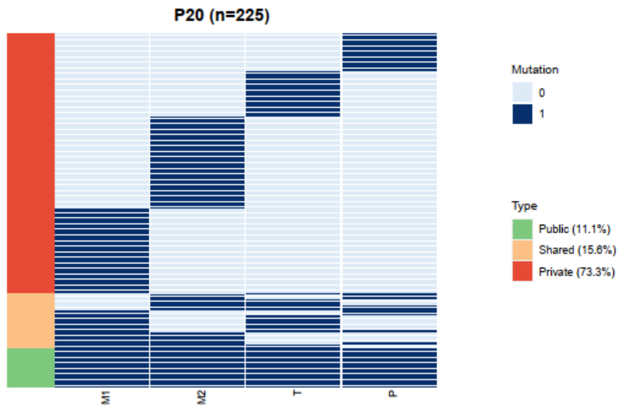
P20 (n=225)
M1
M2
T
P
Mutation
0
1
Type
Public (11.1%)
Shared (15.6%)
Private (73.3%)

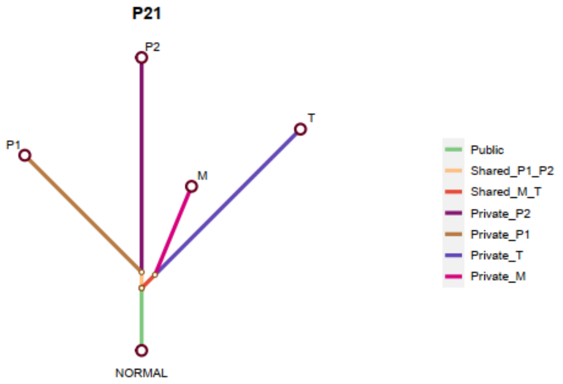
P21
P2
T
P1
M
NORMAL
Public
Shared_P1_P2
Shared_M_T
Private_P2
Private_P1
Private_T
Private_M

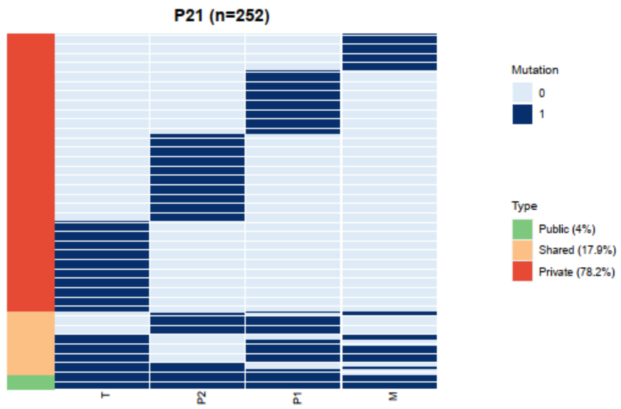
P21 (n=252)
T
P2
P1
M
Mutation
0
1
Type
Public (4%)
Shared (17.9%)
Private (78.2%)

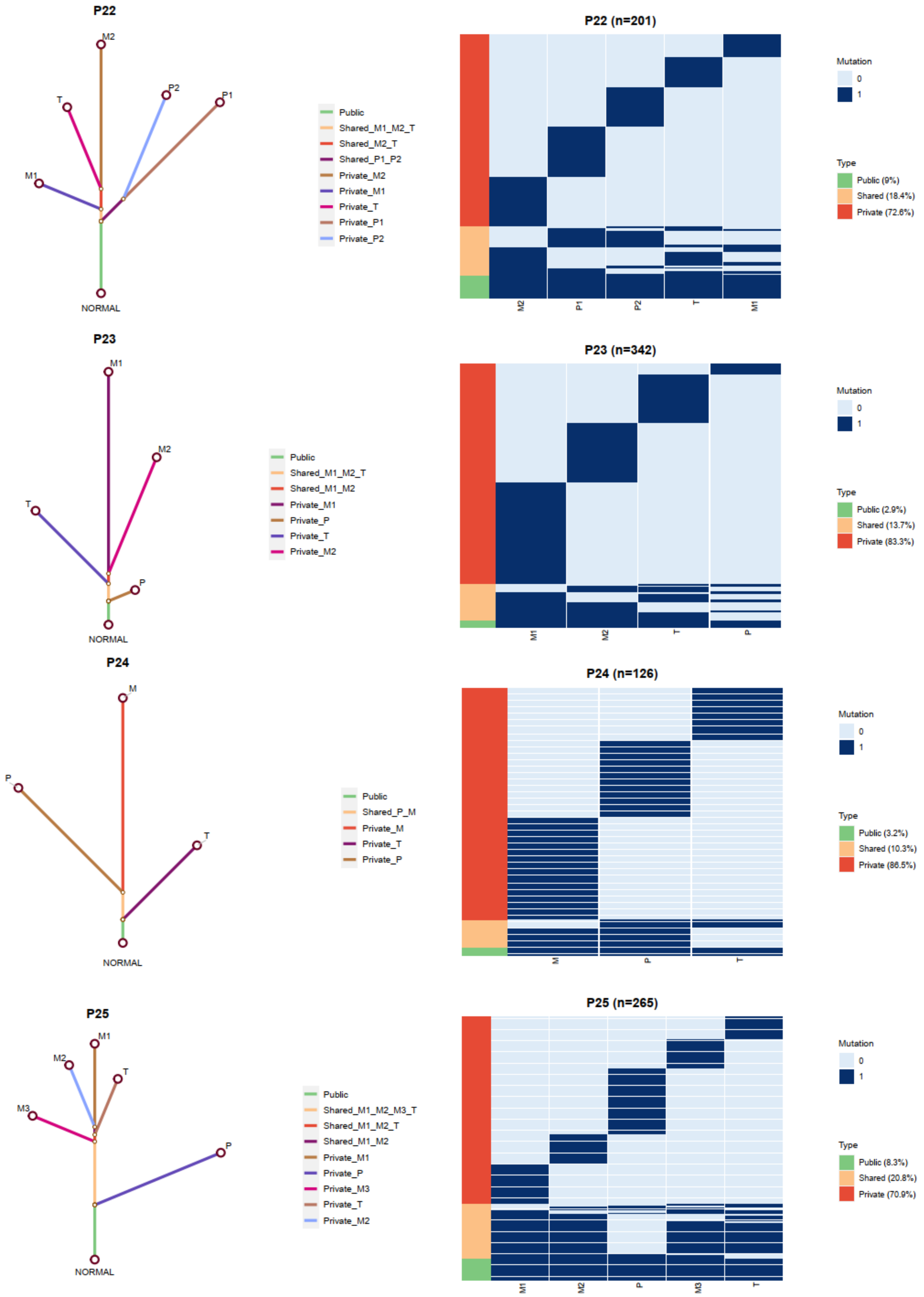
P22
M2
T
P2
P1
M1
NORMAL
Public
Shared_M1_M2_T
Shared_M2_T
Shared_P1_P2
Private_M2
Private_M1
Private_T
Private_P1
Private_P2
P22 (n=201)
M2
P1
P2
T
M1
Mutation
0
1
Type
Public (9%)
Shared (18.4%)
Private (72.6%)
P23
M1
M2
T
P
NORMAL
Public
Shared_M1_M2_T
Shared_M1_M2
Private_M1
Private_P
Private_T
Private_M2
P23 (n=342)
M1
M2
T
P
Mutation
0
1
Type
Public (2.9%)
Shared (13.7%)
Private (83.3%)
P24
M
P
T
NORMAL
Public
Shared_P_M
Private_M
Private_T
Private_P
P24 (n=126)
M
P
T
Mutation
0
1
Type
Public (3.2%)
Shared (10.3%)
Private (86.5%)
P25
M1
M2
T
M3
P
NORMAL
Public
Shared_M1_M2_M3_T
Shared_M1_M2_T
Shared_M1_M2
Private_M1
Private_P
Private_M3
Private_T
Private_M2
P25 (n=265)
M1
M2
P
M3
T
Mutation
0
1
Type
Public (8.3%)
Shared (20.8%)
Private (70.9%)

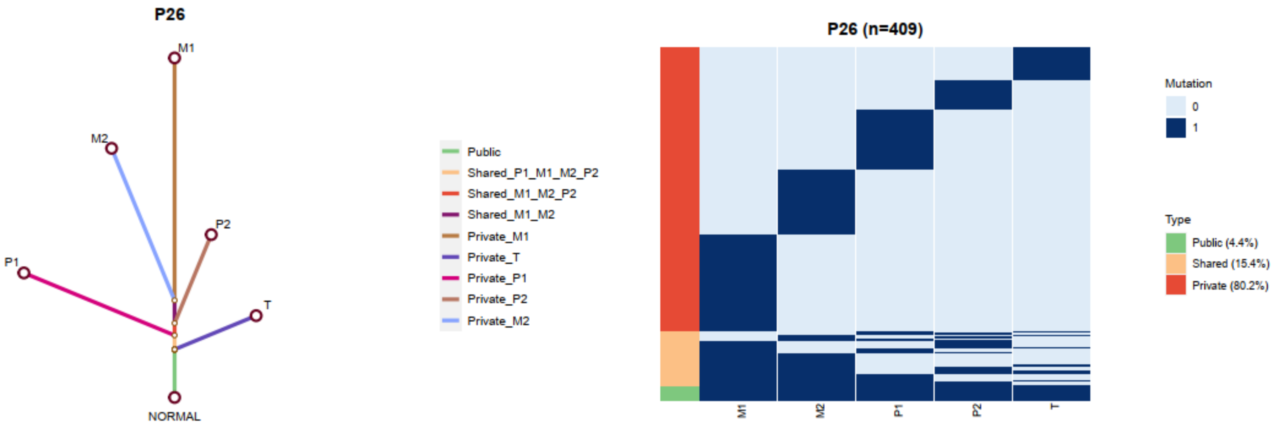
P26
M1
M2
P2
P1
T
NORMAL
Public
Shared_P1_M1_M2_P2
Shared_M1_M2_P2
Shared_M1_M2
Private_M1
Private_T
Private_P1
Private_P2
Private_M2
P26 (n=409)
M1
M2
P1
P2
T
Mutation
0
1
Type
Public (4.4%)
Shared (15.4%)
Private (80.2%)

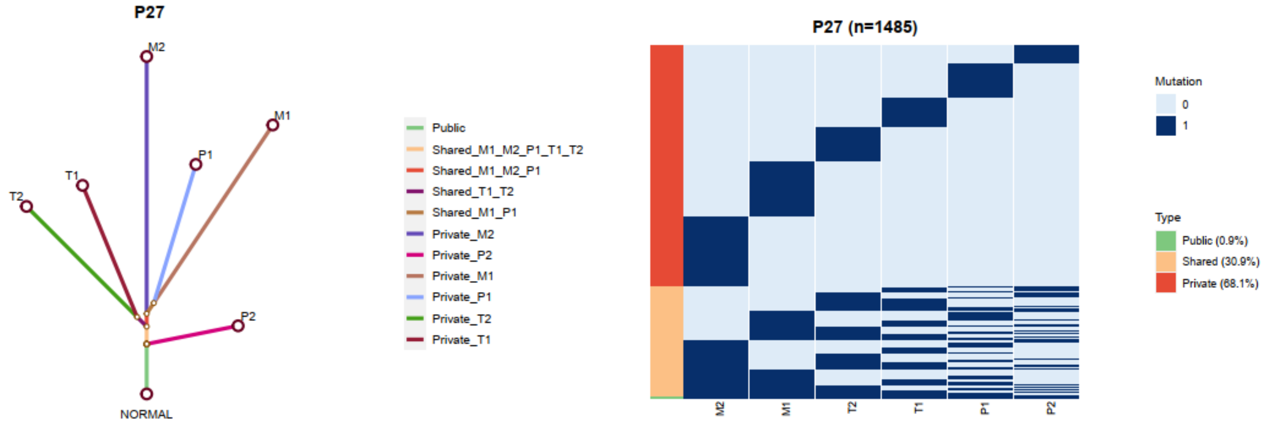
P27
M2
M1
P1
T1
T2
P2
NORMAL
Public
Shared_M1_M2_P1_T1_T2
Shared_M1_M2_P1
Shared_T1_T2
Shared_M1_P1
Private_M2
Private_P2
Private_M1
Private_P1
Private_T2
Private_T1
P27 (n=1485)
M2
M1
T2
T1
P1
P2
Mutation
0
1
Type
Public (0.9%)
Shared (30.9%)
Private (68.1%)

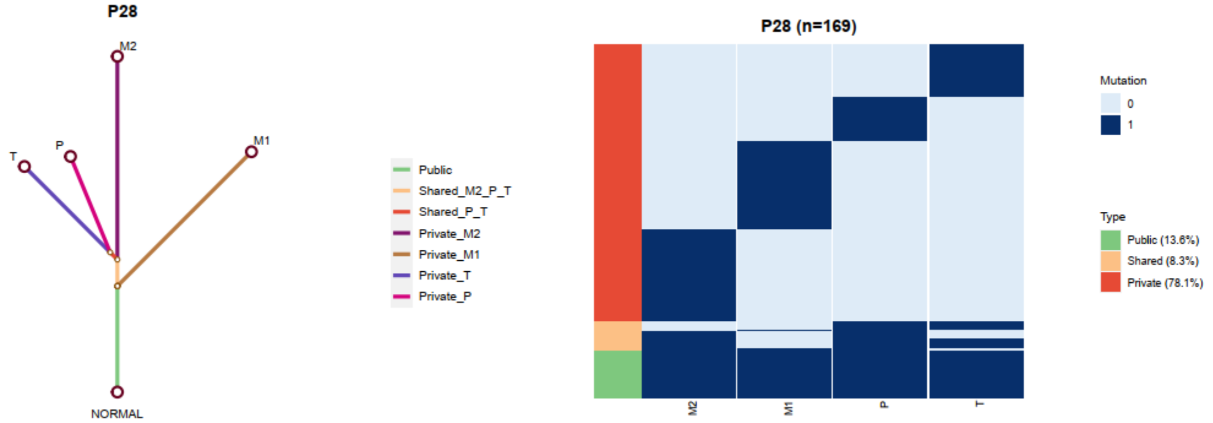
P28
M2
P
T
M1
NORMAL
Public
Shared_M2_P_T
Shared_P_T
Private_M2
Private_M1
Private_T
Private_P
P28 (n=169)
M2
M1
P
T
Mutation
0
1
Type
Public (13.6%)
Shared (8.3%)
Private (78.1%)

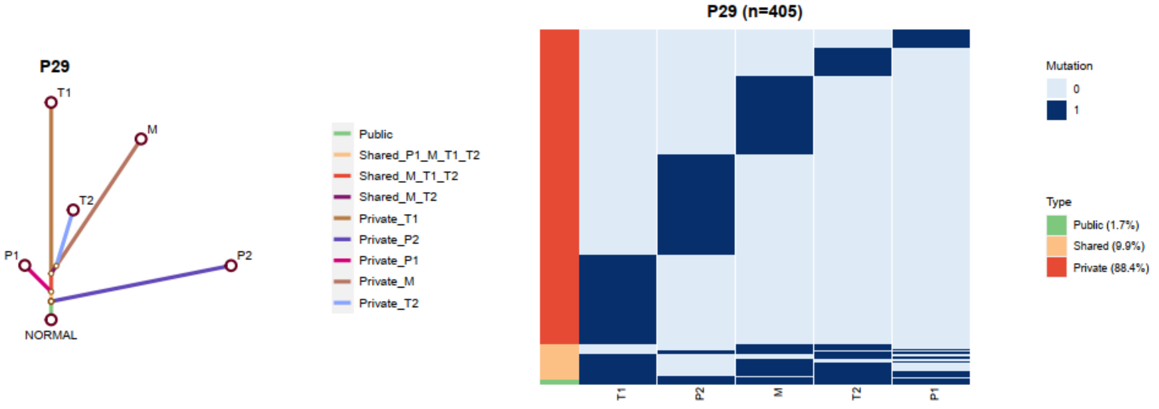
P29
T1
M
T2
P1
P2
NORMAL
Public
Shared_P1_M_T1_T2
Shared_M_T1_T2
Shared_M_T2
Private_T1
Private_P2
Private_P1
Private_M
Private_T2
P29 (n=405)
T1
P2
M
T2
P1
Mutation
0
1
Type
Public (1.7%)
Shared (9.9%)
Private (88.4%)

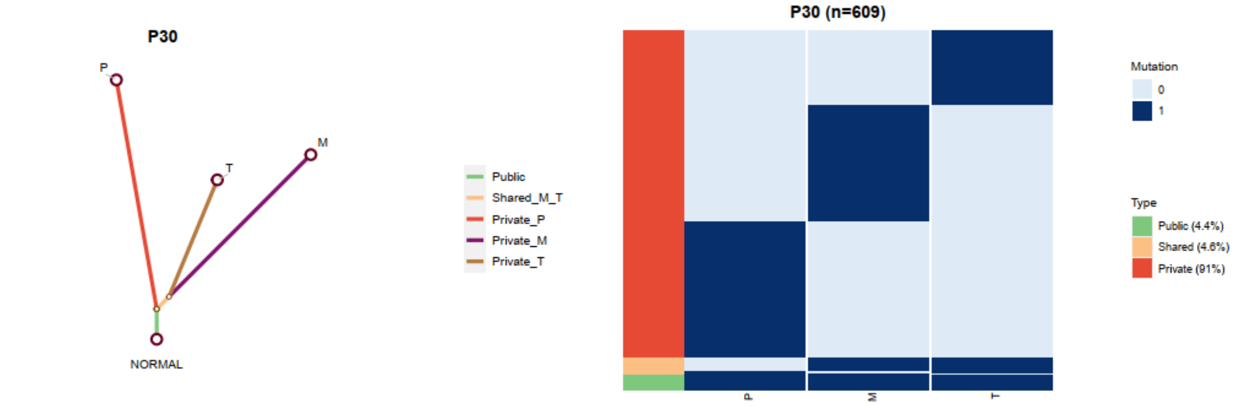
P30
P
T
M
NORMAL
Public
Shared_M_T
Private_P
Private_M
Private_T
P30 (n=609)
P
M
T
Mutation
0
1
Type
Public (4.4%)
Shared (4.6%)
Private (91%)

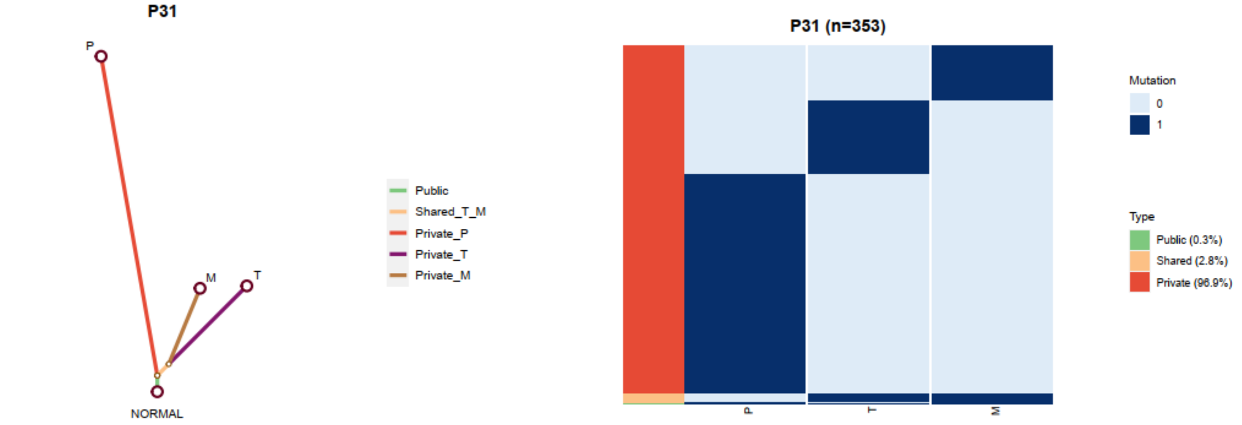
P31
P
M
T
NORMAL
Public
Shared_T_M
Private_P
Private_T
Private_M
P31 (n=353)
P
T
M
Mutation
0
1
Type
Public (0.3%)
Shared (2.8%)
Private (96.9%)

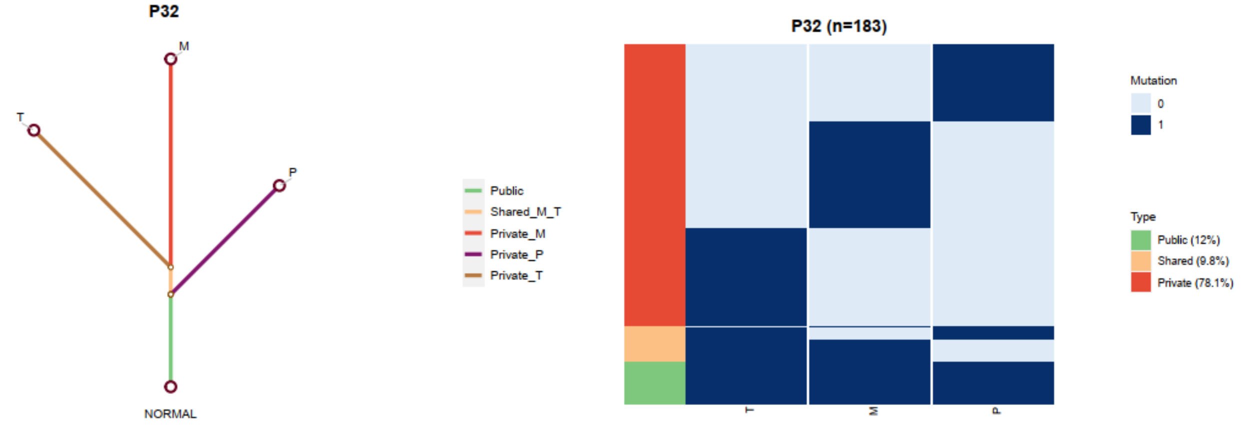
P32
M
T
P
NORMAL
Public
Shared_M_T
Private_M
Private_P
Private_T
P32 (n=183)
T
M
P
Mutation
0
1
Type
Public (12%)
Shared (9.8%)
Private (78.1%)

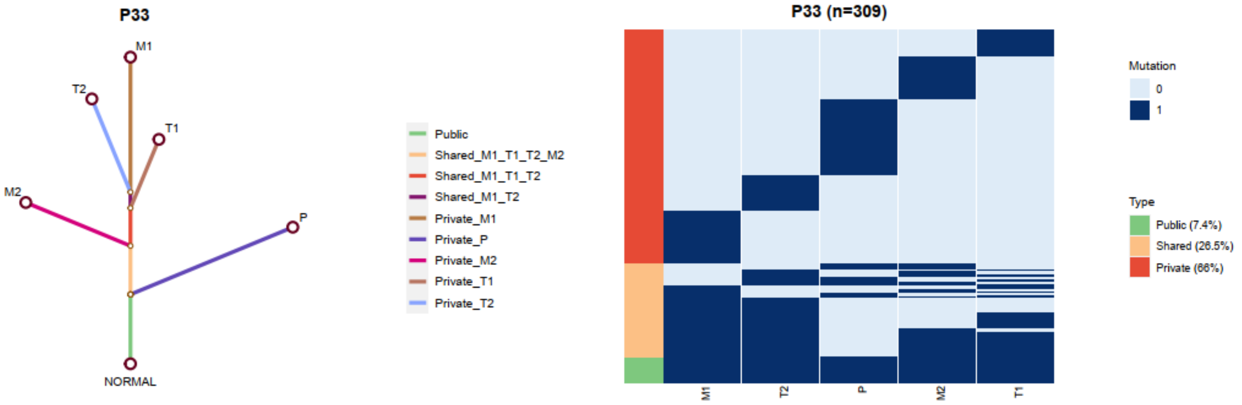
P33
M1
T2
T1
M2
P
NORMAL
Public
Shared_M1_T1_T2_M2
Shared_M1_T1_T2
Shared_M1_T2
Private_M1
Private_P
Private_M2
Private_T1
Private_T2
P33 (n=309)
M1
T2
P
M2
T1
Mutation
0
1
Type
Public (7.4%)
Shared (26.5%)
Private (66%)

Figure S3. Kaplan-Meier estimates of OS (upper) and DFS (lower) stratified by PT grading in (A) Training cohort, and (B) China-Validation cohort, and (C) Poland-Validation cohort (only OS). OS, overall survival; DFS, disease-free survival; PT, primary tumor.

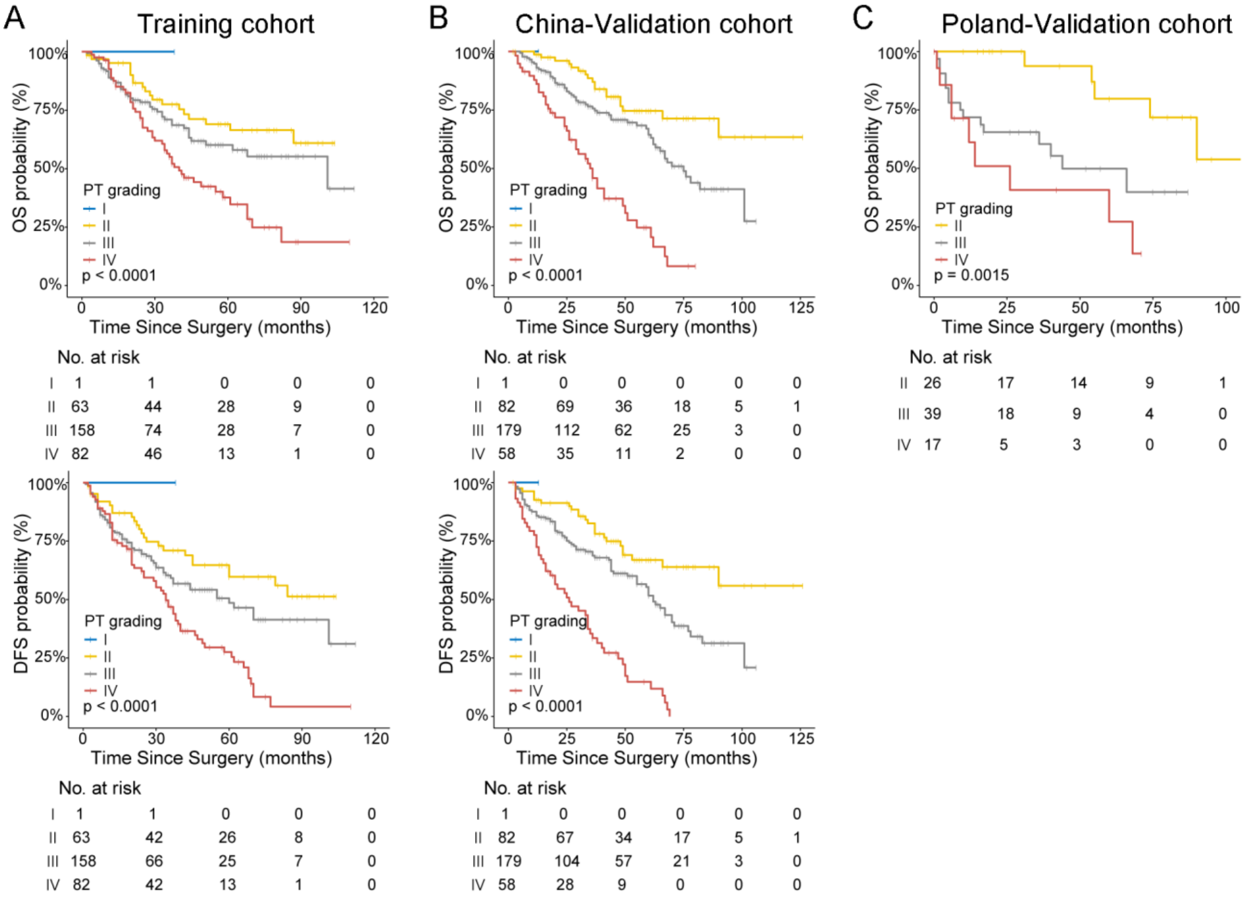

Figure S4. Subgroup analysis and forest plots for OS illustrating multivariate hazard ratios according to VTT grading III vs I-II (blue), and VTT grading IV vs I-II (yellow) in Training cohort. OS, overall survival; VTT, venous tumor thrombus; PT, primary tumor; HR, hazard ratio; CI, confidential interval.

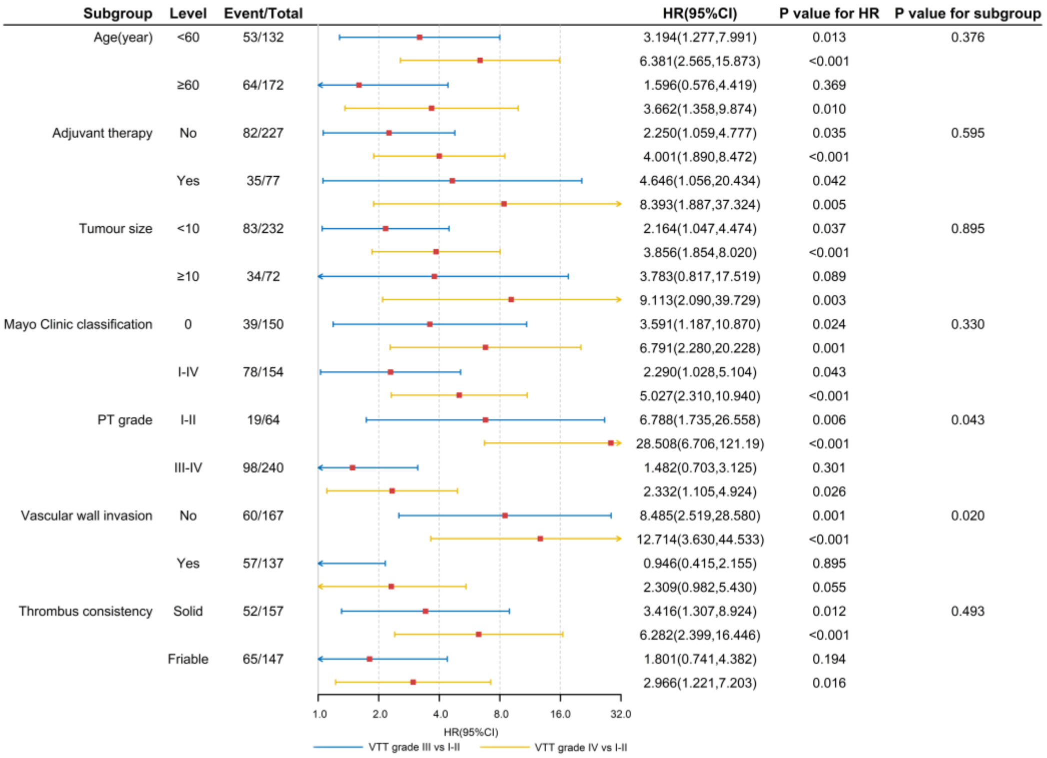

Figure S5. Subgroup analysis and forest plots for DFS illustrating multivariate hazard ratios according to VTT grading III vs I-II (blue), and VTT grading IV vs I-II (yellow) in Training cohort. DFS, disease-free survival; VTT, venous tumor thrombus; PT, primary tumor; HR, hazard ratio; CI, confidential interval.

Subgroup	Level	Event/Total	HR(95%CI)	P value for HR	P value for subgroup
Age(year)	<60	65/132	3.096(1.394,6.876)	0.006	0.965
			5.591(2.507,12.472)	<0.001	
	≥60	87/172	2.704(1.271,5.755)	0.010	
			5.348(2.517,11.366)	<0.001	
Adjuvant therapy	No	109/227	3.147(1.661,5.963)	<0.001	0.926
			6.012(3.228,11.196)	<0.001	
	Yes	43/77	3.390(1.152,9.977)	0.027	
			5.363(1.810,15.891)	0.002	
Tumour size	<10	111/232	2.834(1.513,5.308)	0.001	0.384
			5.032(2.653,9.546)	<0.001	
	≥10	41/72	4.791(1.398,16.415)	0.013	
			9.055(2.676,30.636)	<0.001	
Mayo Clinic classification	0	52/150	5.969(2.062,17.274)	0.001	0.112
			10.251(3.516,29.890)	<0.001	
	I-IV	100/154	1.985(1.045,3.770)	0.036	
			3.946(2.107,7.389)	<0.001	
PT grade	I-II	24/64	4.552(1.605,12.913)	0.004	0.260
			13.979(4.289,45.557)	<0.001	
	III-IV	128/240	2.006(1.036,3.883)	0.039	
			3.103(1.589,6.061)	0.001	
Vascular wall invasion	No	77/167	6.899(2.817,16.894)	<0.001	0.070
			10.914(4.388,27.145)	<0.001	
	Yes	75/137	1.368(0.675,2.770)	0.384	
			2.407(1.136,5.099)	0.022	
Thrombus consistency	Solid	67/157	3.589(1.683,7.654)	0.001	0.645
			6.719(3.224,14.004)	<0.001	
	Friable	85/147	2.548(1.118,5.807)	0.026	
			4.872(2.147,11.054)	<0.001	

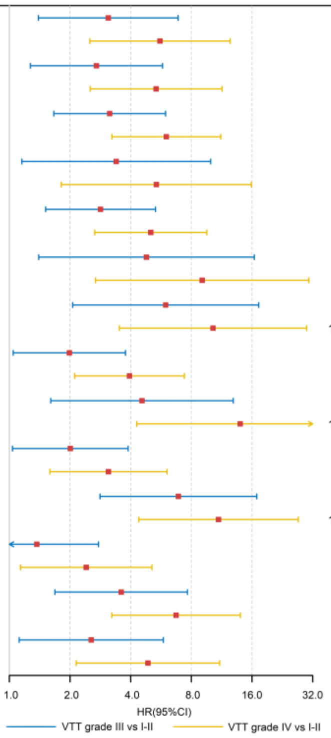

Figure S6. Subgroup analysis and forest plots for OS illustrating multivariate hazard ratios according to VTT grading III vs I-II (blue), and VTT grading IV vs I-II (yellow) in China-Validation cohort. OS, overall survival; VTT, venous tumor thrombus; PT, primary tumor; HR, hazard ratio; CI, confidential interval.

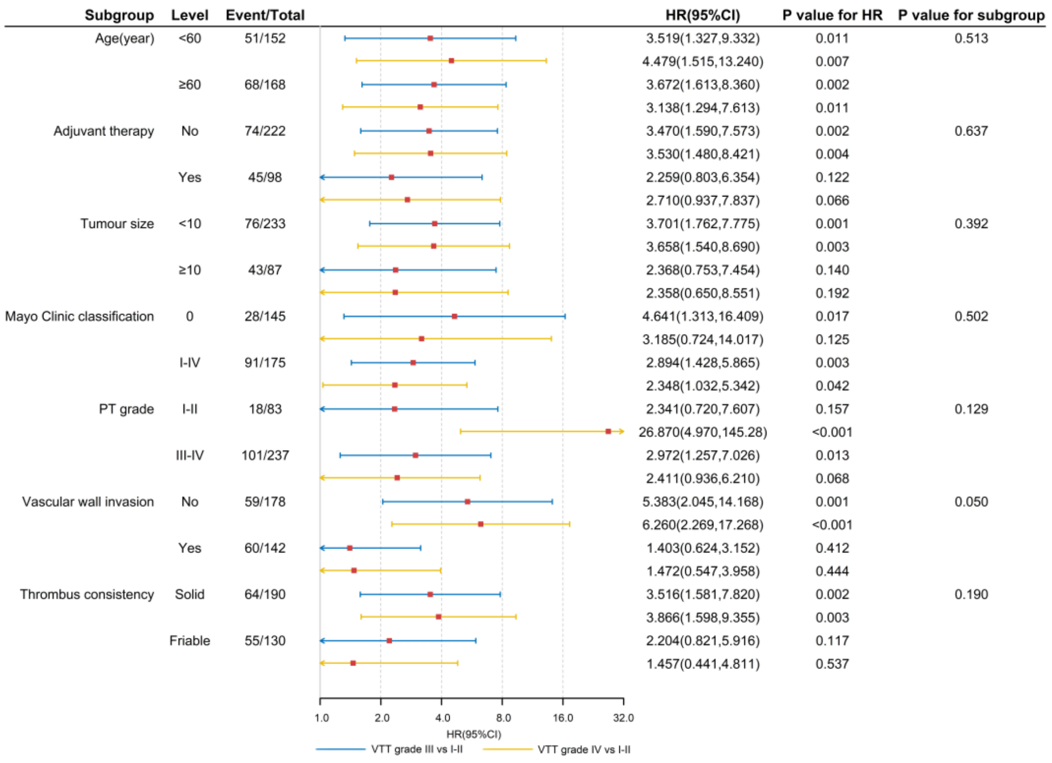

Figure S7. Subgroup analysis and forest plots for DFS illustrating multivariate hazard ratios according to VTT grading III vs I-II (blue), and VTT grading IV vs I-II (yellow) in China-Validation cohort. DFS, disease-free survival; VTT, venous tumor thrombus; PT, primary tumor; HR, hazard ratio; CI, confidential interval.

Subgroup	Level	Event/Total	HR(95%CI)	P value for HR	P value for subgroup
Age(year)	<60	69/152	4.011(1.649,9.757)	0.002	0.169
			4.949(1.797,13.628)	0.002	
	≥60	83/168	3.437(1.692,6.983)	0.001	
			3.321(1.510,7.303)	0.003	
Adjuvant therapy	No	94/222	4.281(2.143,8.550)	<0.001	0.320
			5.271(2.483,11.189)	<0.001	
	Yes	58/98	2.388(0.946,6.029)	0.065	
			3.369(1.238,9.168)	0.017	
Tumour size	<10	101/233	3.834(2.020,7.277)	<0.001	0.696
			3.706(1.695,8.101)	0.001	
	≥10	51/87	4.909(1.421,16.954)	0.012	
			5.890(1.593,21.775)	0.008	
Mayo Clinic classification	0	46/145	3.968(1.354,11.626)	0.012	0.007
			7.199(2.255,22.989)	0.001	
	I-IV	106/175	3.284(1.706,6.322)	<0.001	
			3.075(1.506,6.282)	0.002	
PT grade	I-II	24/83	4.650(1.734,12.468)	0.002	0.374
			9.427(2.270,39.151)	0.002	
	III-IV	128/237	3.595(1.626,7.945)	0.002	
			3.392(1.434,8.024)	0.005	
Vascular wall invasion	No	77/178	5.380(2.370,12.217)	<0.001	0.054
			6.526(2.753,15.465)	<0.001	
	Yes	75/142	2.014(0.944,4.296)	0.070	
			2.299(0.963,5.486)	0.061	
Thrombus consistency	Solid	78/190	3.847(1.899,7.790)	<0.001	0.248
			5.077(2.329,11.068)	<0.001	
	Friable	74/130	2.674(1.105,6.476)	0.029	
			2.851(1.080,7.524)	0.034	

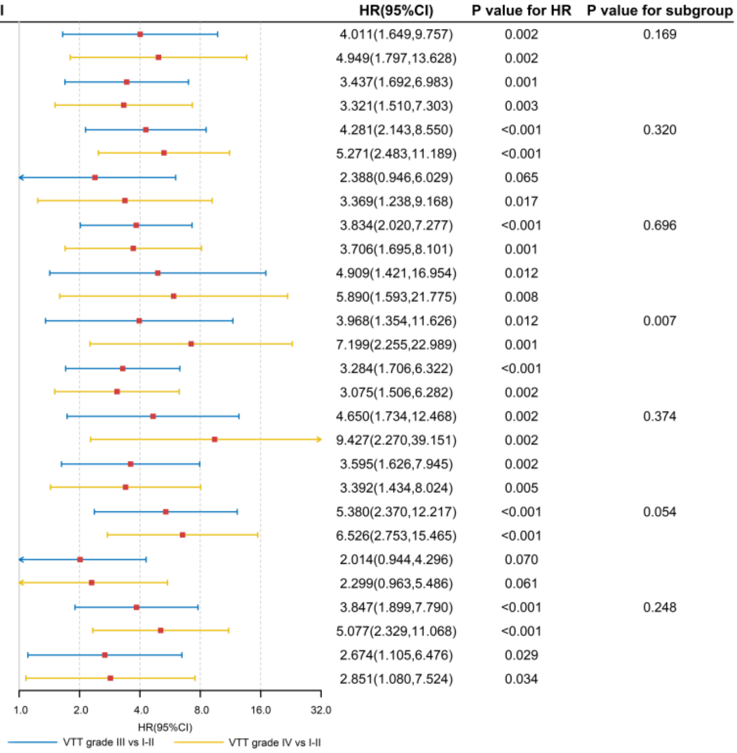

Figure S8. Calibration plot of the TT-GPS score for predicting 5-year OS (upper) and 5-year DFS (lower) in (A) Training cohort, (B) China-Validation cohort, and (C) Poland-Validation cohort. OS, overall survival; DFS, disease-free survival; TT-GPS, VTT height, VTT Grading, Perinephric fat invasion, Sarcomatoid differentiation in PT; SSIGN, the Mayo Clinic Stage, Size, Grade and Necrosis; UISS, the University of California Los Angeles Integrated Staging System; GRANT, the GRade, Age, Nodes and Tumor.

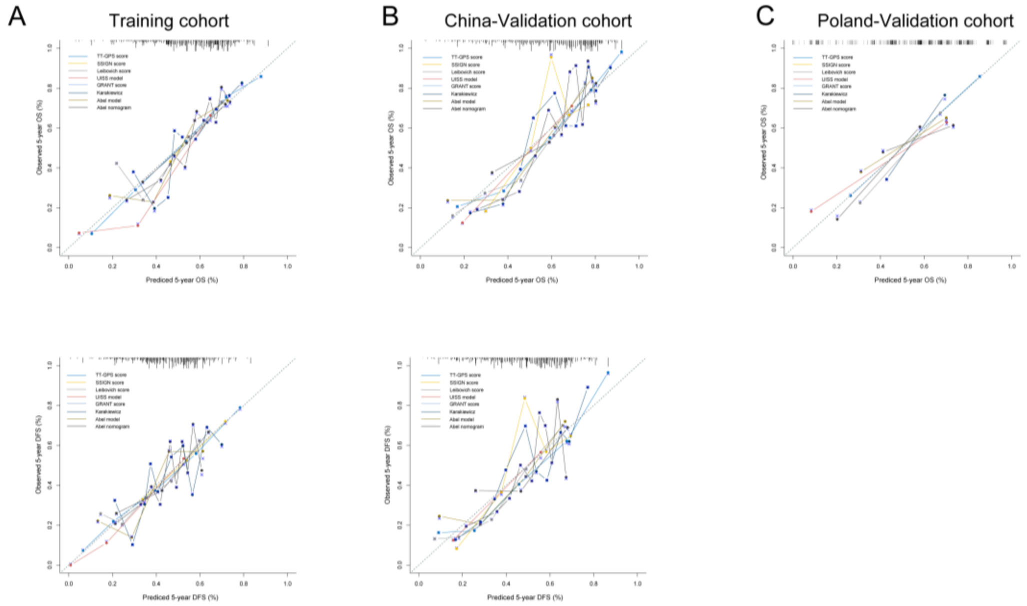

Figure S9. Decision curve analysis of 5-year death risk (upper) and 5-year relapse risk(lower) for the TT-GPS score and other prognostic models in (A) Training cohort, (B) China-Validation cohort, and (C) Poland-Validation cohort. The x-axis represents potential thresholds for 5-year death risk (upper) and 5-year relapse risk(lower), and the y axis represents the net benefit of using the model to risk stratify patients. For instance, in Training cohort with 57% 5-year OS, for a decision threshold of 50% 5-year risk of death, compared with not using any model the TT-GPS score would identify 18 additional true deaths within 5 years per 100 subjects, without increasing the number of false positive predictions. Not using a model assumed that all patients have the same risk and all patients either intervened at all (bold grey solid line) or not at all (bold black solid line). TT-GPS, VTT height, VTT Grading, Perinephric fat invasion, Sarcomatoid differentiation in PT; SSIGN, the Mayo Clinic Stage, Size, Grade and Necrosis; UISS, the University of California Los Angeles Integrated Staging System; GRANT, the GRade, Age, Nodes and Tumor.

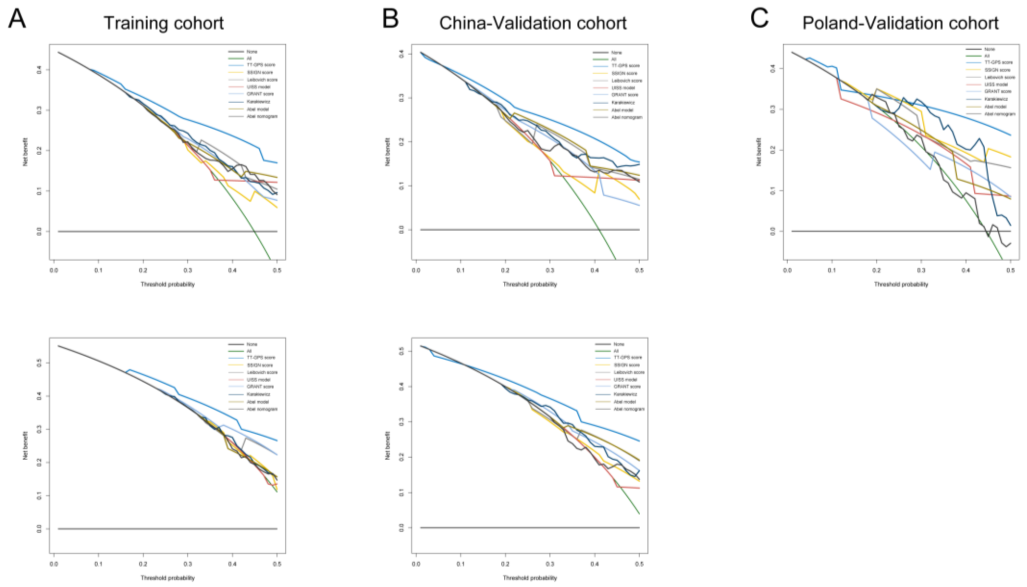

Table S1. Clinical characteristics of 33 ccRCC patients subjected to multi-region WES.

Subject	Age	Gender	Tumor side	Thrombus level	Size of primary tumor (mm)	Nodal involvement	Metastatic disease	Site of metastasis	Histology
P01	64	F	R	0	112	0	Synchronous	Adrenal Gland	Clear cell
P02	67	M	R	3	80	1	Synchronous	Adrenal Gland	Clear cell
P03	54	M	L	0	150	0	Metachronous	Lung	Clear cell
P04	60	M	R	1	95	0	Synchronous	Brain	Clear cell
P05	70	M	L	2	50	0	Metachronous	Lung	Clear cell
P06	66	M	L	0	70	0	Metachronous	Chest wall	Clear cell
P07	33	M	R	0	80	0	Metachronous	Retroperitoneum, Colon	Clear cell
P08	57	M	L	0	110	1	Synchronous	Bone	Clear cell
P09	67	M	L	0	80	0	Metachronous	Lung	Clear cell
P10	55	F	R	2	160	1	Metachronous	Lung	Clear cell
P11	60	M	L	0	53	0	Metachronous	Lung	Clear cell
P12	59	M	R	1	70	1	Synchronous	Vagina	Clear cell
P13	61	M	R	0	40	1	Synchronous	Bone	Clear cell
P14	58	M	L	2	140	0	Metachronous	Brain	Clear cell
P15	61	M	R	2	100	1	Synchronous	Liver	Clear cell
P16	53	M	L	0	60	0	Metachronous	Bone	Clear cell
P17	47	M	L	1	75	0	Metachronous	Bone	Clear cell
P18	46	M	L	0	50	0	Metachronous	Liver	Clear cell
P19	54	M	R	0	85	0	Metachronous	Bone	Clear cell
P20	59	F	L	1	60	1	Metachronous	Bone, Adrenal Gland	Clear cell
P21	71	F	R	0	60	0	Metachronous	Bone	Clear cell
P22	69	M	L	0	90	0	Metachronous	Lung	Clear cell
P23	68	M	L	3	80	0	Metachronous	Adrenal Gland	Clear cell
P24	61	M	L	0	80	0	Metachronous	Brain, Liver	Clear cell
P25	50	M	L	0	75	1	Synchronous	Bone	Clear cell
P26	63	M	L	0	85	0	Metachronous	Brain	Clear cell

P27	66	M	R	3	120	0	Metachronous	Lung, Adrenal Gland	Clear cell
P28	48	F	L	4	110	0	Metachronous	Bone	Clear cell
P29	71	F	R	3	100	0	Metachronous	Lung	Clear cell
P30	47	M	R	1	70	0	Metachronous	Adrenal Gland	Clear cell
P31	49	M	L	3	90	1	Metachronous	Bone	Clear cell
P32	53	M	R	0	60	0	Metachronous	Bone	Clear cell
P33	53	M	L	0	90	1	Metachronous	Bone	Clear cell

Table S2. The clinicopathologic characteristics of patients in indicated cohorts.

Variables	Training cohort (n = 304)	China-Validation cohort (n =320)	Poland-Validation cohort (n = 82)	*P*
Age (years)				0.002
Mean (SD)	60.3 (12.0)	59.4 (10.9)	64.5 (10.3)	
Median (IQR)	61.00 (53.0-69.0)	60.00 (53.0-67.0)	65.00 (59.0-71.0)	
Gender				0.217
Female	112 (36.8)	111 (34.7)	37 (45.1)	
Male	192 (63.2)	209 (65.3)	45 (54.9)	
BMI (kg/m^2)				0.273
Mean (SD)	23.8 (2.9)	24.1 (3.1)	NA	
Median (IQR)	23.4 (21.6-25.8)	23.4 (21.8-26.2)	NA	
Hospital stays (days)				0.261
Mean (SD)	14.8 (8.5)	15.0 (6.0)	14.7 (4.9)	
Median (IQR)	13.0 (8.5-18.0)	14.0 (11.0-18.0)	13.0 (12.0-17.0)	
Hematuria				0.283
No	212 (69.7)	204 (63.8)	55 (67.1)	
Yes	92 (30.3)	116 (36.3)	27 (32.9)	
Osphyalgia				0.536
No	215 (70.72)	237 (74.1)	62 (75.6)	
Yes	89 (29.28)	83 (25.9)	20 (24.4)	
Hypertension				0.453
No	201 (66.1)	223 (69.7)	52 (63.4)	
Yes	103 (33.9)	97 (30.3)	30 (36.6)	
Diabetes				0.004
No	262 (86.2)	259 (80.9)	58 (70.7)	
Yes	42 (13.8)	61 (19.1)	24 (29.3)	
Serum creatinine (μmol/L)				0.154
Mean (SD)	90.1 (59.8)	87.6 (50.3)	NA	

Median (IQR)	83.6 (71.7-103-1)	81.0 (66.0-101.0)	NA	
Serum albumin (g/L)				0.854
Mean (SD)	37.2 (5.1)	27.2 (5.4)	NA	
Median (IQR)	37.4 (33.9-40.8)	37.6 (34.0-41.0)	NA	
Serum hemoglobin (g/L)				0.028
Mean (SD)	112.4 (22.7)	116.5 (21.9)	NA	
Median (IQR)	113.5 (96.0-130.0)	117.0 (100.0-132.0)	NA	
NLR (neu/lym)				0.173
Mean (SD)	4.3 (2.7)	4.6 (2.8)	NA	
Median (IQR)	3.4 (2.2-5.9)	3.5 (2.4-6.5)	NA	
Surgical approach				<0.001
Open	162 (53.3)	177 (55.3)	78 (95.1)	
Laparoscopic	142 (46.7)	143 (44.7)	4 (4.9)	
Surgical time (h)				<0.001
Mean (SD)	4.5 (1.8)	5.0 (1.8)	NA	
Median (IQR)	4.1 (3.2-5.7)	4.6 (3.7-5.9)	NA	
Blood transfusion				<0.001
No	147 (48.4)	172 (53.8)	75 (91.5)	
Yes	157 (51.6)	148 (46.3)	7 (8.5)	
Adjuvant Therapy				0.125
No	227 (74.7)	222 (69.4)	65 (79.3)	
Yes	77 (25.3)	98 (30.6)	17 (20.7)	
Tumor side				0.866
Left	140 (46.0)	141 (44.0)	36 (43.9)	
Right	164 (54.0)	179 (56.0)	46 (56.1)	
Tumor size (cm)				0.233
Mean (SD)	8.0 (3.0)	8.2 (3.3)	8.7 (3.5)	
Median (IQR)	7.7 (5.7-9.9)	8.2 (6.2-10.2)	8.3 (6.0-11.0)	
Thrombus level[a]				<0.001
0	150 (49.3)	145 (45.3)	69 (84.2)	
I	66 (21.7)	64 (20.0)	2 (2.4)	

II	48 (15.8)	59 (18.4)	4 (4.9)	
III	23 (7.6)	39 (12.2)	7 (8.5)	
IV	17 (5.6)	13 (4.1)	0 (0.0)	
Pathological stage				<0.001
T3a	150 (49.3)	145 (45.3)	69 (84.2)	
T3b	127 (41.8)	156 (48.7)	11 (13.4)	
T3c	13 (4.3)	13 (4.1)	0 (0.0)	
T4	14 (4.6)	6 (1.9)	2 (2.4)	
WHO/ISUP grading in PT				0.023
I	1 (0.3)	1 (0.3)	0 (0.0)	
II	63 (20.7)	82 (25.6)	26 (31.7)	
III	158 (52.0)	179 (56.0)	39 (47.6)	
IV	82 (27.0)	58 (18.1)	17 (20.7)	
Tumor necrosis in PT				0.031
No	157 (51.6)	135 (42.2)	44 (53.7)	
Yes	147 (48.4)	185 (57.8)	38 (46.3)	
Sarcomatoid features in PT				0.494
No	258 (84.9)	268 (83.8)	73 (89.0)	
Yes	46 (15.1)	52 (16.2)	9 (11.0)	
Rhabdoid features in PT				0.045
No	262 (86.2)	292 (91.2)	77 (93.9)	
Yes	42 (13.8)	28 (8.8)	5 (6.1)	
Perirenal fat invasion				0.604
No	214 (70.4)	222 (69.4)	53 (64.6)	
Yes	90 (29.6)	98 (30.6)	29 (35.4)	
WHO/ISUP grading in VTT				0.154
I	14 (4.6)	21 (6.5)	0 (0.00)	
II	68 (22.4)	72 (22.5)	20 (24.4)	
III	127 (41.8)	147 (46.0)	37 (45.1)	
IV	95 (31.2)	80 (25.0)	25 (30.5)	
Tumor necrosis in VTT				0.483

No	201 (66.1)	220 (68.8)	NA	
Yes	103 (33.9)	100 (31.2)	NA	
Sarcomatoid features in VTT				0.772
No	274 (90.1)	291 (90.9)	76 (92.7)	
Yes	30 (9.9)	29 (9.1)	6 (7.3)	
Rhabdoid features in VTT				0.001
No	277 (91.1)	302 (94.4)	67 (81.7)	
Yes	27 (8.9)	18 (5.6)	15 (18.3)	
Vascular wall invasion				0.862
No	167 (54.9)	178 (55.6)	NA	
Yes	137 (45.1)	142 (44.4)	NA	
Thrombus consistency				0.052
Friable	147 (48.4)	190 (59.4)	NA	
Solid	157 (51.6)	130 (40.6)	NA	
5-year OS probability (%)			55.6	
5-year DFS probability (%)			NA	

NOTE. Data are presented as No. (%) unless indicated otherwise.

[a]According to the Mayo Clinic Classification

Abbreviations: NLR, neutrophil to lymphocyte ratio; PT, primary tumor; VTT, venous tumor thrombus; OS, overall survival; DFS, disease-free survival; NA, not available.

P values were calculated by ANOVA or Kruskal-Wallis tests for continuous variables, Chi-square test for categorical variables and Cochran-Mantel-Haenszel(CMH) Chi-square test for ordinal variables.

Table S3. Univariable and Multivariable Cox Regression Analysis of Variables with OS in indicated cohorts.

Variables	Comparison	Training cohort (n = 304)				China-Validation cohort (n = 320)				Poland-Validation cohort (n = 82)			
		Univariable analysis		Multivariable analysis		Univariable analysis		Multivariable analysis		Univariable analysis		Multivariable analysis	
		HR (95% CI)	*P*	HR (95% CI)	*P*	HR (95% CI)	*P*	HR (95% CI)	*P*	HR (95% CI)	*P*	HR (95% CI)	*P*
Age	Per year	0.997 (0.983-1.012)	0.721			1.010 (0.993-1.028)	0.252			0.966 (0.933-1.001)	0.055		
Gender	Female vs. male	1.322 (0.917- 1.904)	0.134			1.233 (0.852-1.784)	0.267			0.812 (0.383-1.722)	0.588		
BMI	Per kg/m^2	1.020 (0.957- 1.087)	0.547			0.956 (0.900-1.015)	0.143						
Hospital stays	Per day	1.021 (1.003-1.039)	0.019			1.020 (0.994-1.046)	0.130			1.017 (0.928-1.114)	0.725		
Hematuria	Yes vs. no	0.936 (0.630-1.392)	0.745			1.076 (0.743-1.559)	0.699			2.275 (1.080-4.794)	0.031		
Osphyalgia	Yes vs. no	1.019 (0.686-1.514)	0.925			1.195 (0.802-1.780)	0.381			1.630 (0.708-3.754)	0.251		
Hypertension	Yes vs. no	1.152 (0.785-1.691)	0.468			0.827 (0.551-1.241)	0.360			1.087 (0.505-2.341)	0.831		
Diabetes	Yes vs. no	1.479 (0.913-2.398)	0.112			1.483 (0.976-2.256)	0.065			1.045 (0.461-2.369)	0.917		
Serum creatinine	Per μmol/L	1.002 (0.999-1.004)	0.083			1.000 (0.997-1.004)	0.887						
Serum albumin	Per g/L	0.993 (0.958-1.029)	0.692			0.974 (0.943-1.007)	0.121						
Serum hemoglobin	Per g/L	0.990 (0.982-0.998)	0.016			0.993 (0.984-1.001)	0.086						
NLR	Per neu/lym	1.082 (1.020-1.147)	0.008			1.026 (0.966-1.089)	0.402						
Surgical approach	Laparoscopic vs. Open	0.967(0.671-1.392)	0.856			0.828 (0.573-1.195)	0.313			0.444 (0.060-3.277)	0.426		
Surgical time	Per hour	1.147 (1.041-1.263)	0.005			1.079 (0.986-1.181)	0.097						
Blood transfusion	Yes vs. no	1.463 (1.010-2.118)	0.044			2.182 (1.500-3.173)	< 0.001			1.762 (0.531-5.845)	0.354		
Adjuvant Therapy	Yes vs. no	1.394 (0.938-2.072)	0.101			1.509 (1.042-2.186)	0.030			1.111 (0.474-2.603)	0.809		
Tumor side	Right vs. Left	0.999 (0.694-1.439)	0.997			0.684 (0.476-0.985)	0.041			0.890 (0.414-1.914)	0.765		
Tumor size	Per cm	1.026 (0.963-1.093)	0.425			1.076 (1.022-1.133)	0.005			1.070 (0.966-1.185)	0.192		
Thrombus	0	reference		reference		reference		reference		reference		reference	

level[a]	I	1.671 (1.038-2.691)	0.035	1.062 (0.643-1.755)	0.815	1.854 (1.068-3.249)	0.028	1.469 (0.832-2.595)	0.185	1.349 (0.178-10.201)	0.772	2.259 (0.252-20.249)	0.467
	II	2.092 (1.228-3.562)	0.007	1.728 (1.009-2.961)	0.046	2.570 (1.528-4.322)	< 0.001	1.781 (1.043-3.041)	0.034	8.432 (2.683-26.494)	< 0.001	3.239 (0.920-11.401)	0.067
	III	3.041 (1.697-5.447)	< 0.001	1.712 (0.931-3.146)	0.084	3.631 (2.150-6.133)	< 0.001	2.098 (1.202-3.665)	0.009	8.735 (3.485-21.899)	< 0.001	14.045 (4.406-44.772)	< 0.001
	IV	3.713 (1.893-7.282)	< 0.001	2.734 (1.373-5.444)	0.004	5.409 (2.680-10.915)	< 0.001	3.026 (1.467-6.241)	0.003	N.A.	N.A.	NA	NA
Pathological stage	T3a	reference				reference				reference			
	T3b	1.855 (1.235-2.785)	0.003			2.433 (1.574-3.759)	< 0.001			6.288 (2.789-14.177)	< 0.001		
	T3c	3.187 (1.420-7.151)	0.005			5.431 (2.691-10.962)	< 0.001			N.A.	N.A.		
	T4	4.710 (2.507-8.850)	< 0.001			6.251 (2.580-15.143)	< 0.001			4.180 (0.947-18.448)	0.059		
WHO/ISUP grading in PT	I-II	reference		reference		reference		reference		reference		reference	
	III	1.533 (0.899-2.611)	0.116			1.969 (1.161-3.340)	0.012			4.312 (1.414-13.150)	0.010		
	IV	2.690 (1.578-4.588)	< 0.001			5.546 (3.269-10.101)	< 0.001			7.228 (2.175-24.026)	0.001		
Tumor necrosis in PT	Yes vs. no	1.200 (0.834-1.726)	0.326			1.089 (0.757-1.566)	0.645			2.378 (1.116-5.067)	0.025		
Sarcomatoid features in PT	Yes vs. no	3.539 (2.419-5.177)	< 0.001	2.036 (1.305-3.177)	0.002	4.126 (2.805-6.071)	< 0.001	2.277 (1.429-3.628)	< 0.001	5.033 (2.142-11.828)	< 0.001	3.999 (1.330-12.029)	0.014
Rhabdoid features in PT	Yes vs. no	1.653 (1.052-2.597)	0.029			3.063 (1.933-4.854)	< 0.001			2.150 (0.279-16.557)	0.462		
Perirenal fat invasion	Yes vs. no	1.795 (1.243-2.591)	0.002	1.552 (1.056-2.283)	0.025	2.980 (2.070-4.291)	< 0.001	2.296 (1.563-3.373)	< 0.001	2.514 (1.200-5.270)	0.015	2.949 (1.246-6.981)	0.014
WHO/ISUP grading in VTT	I-II	reference		reference		reference		reference		reference		reference	
	III	2.931 (1.537-5.587)	0.001	2.727 (1.416-5.252)	0.003	3.745 (2.037-6.886)	< 0.001	3.041 (1.643-5.626)	< 0.001	5.888 (1.289-26.891)	0.022	3.474 (0.627-19.242)	0.154
	IV	6.903 (3.695-12.896)	< 0.001	4.877 (2.513-9.465)	< 0.001	7.274 (3.929-13.466)	< 0.001	2.524 (1.203-5.297)	0.014	13.648 (2.945-63.254)	< 0.001	8.277 (1.526-44.904)	0.014

Tumor necrosis in VTT	Yes vs. no	0.933 (0.634-1.376)	0.728			1.171 (0.798-1.717)	0.421			N.A.	N.A.
Sarcomatoid features in VTT	Yes vs. no	2.561 (1.619-4.051)	< 0.001			3.823 (2.394-6.106)	< 0.001	1.997 (1.123-3.551)	0.019	1.778 (0.611-5.172)	0.291
Rhabdoid features in VTT	Yes vs. no	2.712 (1.701-4.324)	< 0.001			4.169 (2.480-7.006)	< 0.001			1.432 (0.540-3.800)	0.471
Vascular wall invasion	Yes vs. no	1.363 (0.947-1.960)	0.095			1.531 (1.067-2.195)	0.021			N.A.	N.A.
Thrombus consistency	Friable vs. Solid	1.557 (1.080-2.245)	0.018			1.620 (1.127-2.328)	0.009			N.A.	N.A.

[a]According to the Mayo Clinic Classification

Abbreviations: NLR, neutrophil to lymphocyte ratio; PT, primary tumor; VTT, venous tumor thrombus; HR, hazard ratio; CI, confidential interval; NA, not available; OS, overall survival.

Table S4. Univariable and Multivariable Cox Regression Analysis of Variables with DFS in indicated cohorts.

Variables	Class	Training cohort (n = 304)				China-Validation cohort (n = 320)			
		Univariable analysis		Multivariable analysis		Univariable analysis		Multivariable analysis	
		HR (95% CI)	*P*	HR (95% CI)	*P*	HR (95% CI)	*P*	HR (95% CI)	*P*
Age	Per year	1.000 (0.987-1.014)	0.957			1.000 (0.985-1.016)	0.958		
Gender	Female vs. male	1.172 (0.849-1.618)	0.335			1.200 (0.866-1.664)	0.273		
BMI	Per kg/m^2	1.003 (0.948-1.061)	0.920			0.968 (0.918-1.021)	0.232		
Hospital stays	Per day	1.011 (0.994-1.027)	0.203			1.015 (0.991-1.040)	0.210		
Hematuria	Yes vs. no	1.024(0.724-1.448)	0.895			1.054 (0.759-1.464)	0.753		
Osphyalgia	Yes vs. no	1.070 (0.758-1.510)	0.700			1.150 (0.803-1.646)	0.446		
Hypertension	Yes vs. no	1.131 (0.806-1.589)	0.476			0.690 (0.476-1.000)	0.050		
Diabetes	Yes vs. no	1.237 (0.793-1.930)	0.349			1.137 (0.766-1.687)	0.524		
Serum creatinine	Per μmol/L	1.001 (0.999-1.003)	0.198			1.000 (0.996-1.003)	0.803		
Serum albumin	Per g/L	0.992 (0.961-1.024)	0.615			0.983 (0.955-1.013)	0.261		
Serum hemoglobin	Per g/L	0.990 (0.983-0.997)	0.006			0.993 (0.986-1.001)	0.073		
NLR	Per neu/lym	1.042 (0.988-1.100)	0.129			1.026 (0.973-1.081)	0.348		
Surgical approach	Laparoscopic vs. Open	0.786 (0.568-1.087)	0.146			0.760 (0.547-1.054)	0.100		
Surgical time	Per hour	1.098 (1.007-1.197)	0.035			1.078 (0.993-1.170)	0.072		
Blood transfusion	Yes vs. no	1.606 (1.158-2.226)	0.005			1.937 (1.398-2.684)	<0.001		
Adjuvant Therapy	Yes vs. no	1.411 (0.991-2.010)	0.056			1.571 (1.132-2.180)	0.007		
Tumor side	Right vs. Left	0.973 (0.707-1.338)	0.866			0.657 (0.477-0.906)	0.010		
Tumor size	Per cm	1.025 (0.971-1.083)	0.371			1.058 (1.010-1.108)	0.017		
Thrombus level[a]	0	reference	reference	reference	reference	reference	reference		
	I	1.819 (1.211-2.732)	0.004	1.503 (0.994-2.274)	0.054	1.469 (0.922-2.338)	0.105		
	II	2.290 (1.443-3.634)	<0.001	1.767 (1.106-2.282)	0.017	1.998 (1.287-3.104)	0.002		
	III	3.156 (1.863-5.347)	<0.001	2.175 (1.269-3.729)	0.005	2.449 (1.552-3.864)	<0.001		
	IV	2.964 (1.539-5.708)	0.001	2.573 (1.328-4.986)	0.005	3.426 (1.765-6.652)	<0.001		
Pathological stage	T3a	reference	reference			reference	reference		

	T3b	1.973 (1.390-2.802)	<0.001			1.836 (1.286-2.622)	<0.001		
	T3c	2.373 (1.074-5.246)	0.033			3.431 (1.766-6.662)	<0.001		
	T4	7.347 (4.018-13.434)	<0.001			4.222 (1.799-9.907)	<0.001		
WHO/ISUP grading in PT	I-II	reference	reference	reference	reference	reference	reference	reference	reference
	III	1.693 (1.056-2.714)	0.029			1.926 (1.217-3.047)	0.005		
	IV	2.956 (1.830-4.772)	<0.001			5.686 (3.457-9.353)	<0.001		
Tumor necrosis in PT	Yes vs. no	1.159 (0.842-1.595)	0.365			1.148 (0.831-1.586)	0.401		
Sarcomatoid features in PT	Yes vs. no	2.693 (1.896-3.826)	<0.001			4.248 (3.000-6.017)	<0.001	2.381 (1.581-3.585)	<0.001
Rhabdoid features in PT	Yes vs. no	1.973 (1.335-2.915)	<0.001			2.844 (1.853-4.363)	<0.001		
Perirenal fat invasion	Yes vs. no	1.457 (1.048-2.025)	0.025	1.445 (1.031-2.025)	0.033	2.403 (1.743-3.314)	<0.001	1.940 (1.395-2.698)	<0.001
WHO/ISUP grading in VTT	I-II	reference	reference	reference	reference	reference	reference	reference	reference
	III	3.156 (1.837-5.423)	<0.001	3.184 (1.838-5.513)	<0.001	4.193 (2.426-7.248)	<0.001	3.626 (2.090-6.291)	<0.001
	IV	6.477 (3.800-11.040)	<0.001	5.922 (3.447-10.175)	<0.001	8.086 (4.636-14.103)	<0.001	3.667 (1.931-6.961)	<0.001
Tumor necrosis in VTT	Yes vs. no	0.948 (0.673-1.336)	0.762			1.350 (0.969-1.881)	0.076		
Sarcomatoid features in VTT	Yes vs. no	2.507 (1.648-3.813)	<0.001			3.966 (2.620-6.005)	<0.001	1.735 (1.045-2.878)	0.033
Rhabdoid features in VTT	Yes vs. no	2.768 (1.809-4.235)	<0.001			3.928 (2.392-6.452)	<0.001		
Vascular wall invasion	Yes vs. no	1.420 (1.032-1.953)	0.031			1.465 (1.064-2.016)	0.019		
Thrombus consistency	Friable vs. Solid	1.631 (1.183-2.250)	0.003			1.845 (1.340-2.541)	<0.001		

[a]According to the Mayo Clinic Classification

Abbreviations: NLR, neutrophil to lymphocyte ratio; PT, primary tumor; VTT, venous tumor thrombus; HR, hazard ratio; CI, confidential interval; DFS, disease-free survival.

Table S5. Estimated OS by the TT-GPS score in total patients.

TT-GPS score	No. of patients (%)	Estimated OS				
		Median (95% CI), month	Year 1 (95%CI), %	Year 3 (95%CI), %	Year 5 (95%CI), %	Year 7 (95%CI), %
0	112 (15.86)	NA	98.1 (95.5-100)	96.7 (93.1-100)	94.9 (90.0-100)	92.3 (85.4-99.6)
1	61 (8.64)	NA	94.9 (89.4-100)	85.1 (76.1-95.3)	82.4 (72.4-93.8)	82.4 (72.4-93.8)
2	169 (23.94)	NA	95.6 (92.4-98.8)	87.1 (81.5-93.2)	76.9 (69.1-85.5)	65.0 (54.5-77.5)
3	149 (21.1)	65 (55-NA)	84.0 (78.2-90.2)	66.5 (58.6-75.5)	52.7 (43.0-64.7)	39.2 (28.1-54.8)
4	114 (16.15)	37 (33-46)	89.3 (83.7-95.2)	52.2 (42.9-63.4)	32.9 (24.0-45.1)	13.0 (6.21-27.2)
≥5	101 (14.31)	29 (22-35)	82.0 (74.8-89.9)	36.2 (27.7-47.4)	14.5 (8.65-24.4)	2.64 (4.95-14.1)

Abbreviations: OS, overall survival; CI, confidential interval; NA, not available; TT-GPS, VTT height, VTT Grading, Perinephric fat invasion, Sarcomatoid differentiation in PT.

Table S6. Estimated DFS by the TT-GPS score in total China patients.

TT-GPS score	No. of patients (%)	Estimated DFS				
		Median (95% CI), month	Year 1 (95%CI), %	Year 3 (95%CI), %	Year 5 (95%CI), %	Year 7 (95%CI), %
0	98 (15.71)	NA	96.8 (93.2-100)	95.2 (90.7 100)	95.2 (90.7 100)	92.5 (85.9 99.7)
1	55 (8.81)	NA	89.0 (81.0-97.7)	82.5 (72.7 93.7)	69.4 (56.1 85.9)	65.6 (51.5 83.4)
2	147 (23.56)	83(60-NA)	90.2 (85.4-95.2)	76.0 (68.6 84.3)	57.4 (47.9 68.9)	48.6 (36.9 63.9)
3	130 (20.83)	40 (29-62)	73.8 (66.6-81.7)	53.3 (44.8 63.3)	38.0 (28.6 50.4)	15.6 (7.38 32.9)
4	99 (15.87)	33 (25-42)	77.7 (69.9 86.4)	45.0 (35.6-56.9)	24.5 (16.4 36.6)	3.77 (0.99 14.4)
≥5	95 (15.22)	21 (20-30)	70.5 (61.9-80.3)	30.3 (22.1-41.5)	12.2 (6.87 21.6)	2.43 (0.62 9.54)

Abbreviations: DFS, disease-free survival; CI, confidential interval; NA, not available; TT-GPS, VTT height, VTT Grading, Perinephric fat invasion, Sarcomatoid differentiation in PT.

Table S7. Estimated OS by the TT-GPS risk classification in total patients.

TT-GPS risk classification	No. of patients (%)	Estimated OS				
		Median (95% CI), month	Year 1 (95%CI), %	Year 3 (95%CI), %	Year 5 (95%CI), %	Year 7 (95%CI), %
Low risk (0-2)	342 (48.4)	NA	96.2 (94.2-98.3)	89.9 (86.3-93.5)	83.9 (79.3-88.9)	78.0 (72.1-84.5)
Intermediate risk (3, 4)	263 (37.3)	46 (40-65)	86.3 (82.2-90.6)	59.9 (53.7-66.9)	43.7 (36.8-52.0)	24.8 (17.2-35.9)
High risk (≥ 5)	101 (14.3)	29 (22-35)	82.0 (74.8-89.9)	36.2 (27.7-47.4)	14.5 (8.65-24.4)	2.64 (0.50-14.1)

Abbreviations: OS, overall survival; CI, confidential interval; NA, not available; TT-GPS, VTT height, VTT Grading, Perinephric fat invasion, Sarcomatoid differentiation in PT.

Table S8. Estimated DFS by the TT-GPS risk classification in total China patients.

TT-GPS risk classification	No. of patients (%)	Estimated DFS				
		Median (95% CI), month	Year 1 (95%CI), %	Year 3 (95%CI), %	Year 5 (95%CI), %	Year 7 (95%CI), %
Low risk (0-2)	300 (48.1)	NA	92.0 (89.0-95.2)	83.5 (79.0-88.3)	71.9 (65.8-78.7)	66.5 (59.3-74.7)
Intermediate risk (3, 4)	229 (36.7)	36 (29-44)	75.5 (70.1-81.3)	49.6 (43.1-57.0)	32.2 (25.6-40.5)	8.61 (4.20-17.6)
High risk (≥ 5)	95 (15.2)	21 (20-30)	70.5 (61.9-80.3)	30.3 (22.1-41.5)	12.2 (6.87-21.6)	2.43 (0.62-9.54)

Abbreviations: DFS, disease-free survival; CI, confidential interval; NA, not available; TT-GPS, VTT height, VTT Grading, Perinephric fat invasion, Sarcomatoid differentiation in PT.

Table S9. Subgroup analysis illustrating multivariate hazard ratios and c-index for OS according to the TT-GPS risk classification in Training cohort.

Group	Event/Total	TT-GPS risk classification	HR (95%CI)	c-index (95%CI)
SSIGN score Intermediate risk (4-7)	87/246	Low risk	Reference	0.717 (0.663-0.77)
		Intermediate risk	4.538 (2.592-7.943)	
		High risk	11.336 (5.941-21.630)	
Leibovich score Intermediate risk (3-5)	26/111	Low risk	Reference	0.678 (0.578-0.779)
		Intermediate risk	3.050 (1.310-7.103)	
		High risk	6.085 (1.888-19.610)	
UISS model Intermediate risk	73/259	Low risk	Reference	0.751 (0.699-0.802)
		Intermediate risk	5.195 (2.809-9.608)	
		High risk	15.070 (7.413-30.638)	
GRANT score unfavorable	92/208	Low risk	Reference	0.657 (0.597-0.717)
		Intermediate risk	2.858 (1.587-5.147)	
		High risk	6.526 (3.515-12.115)	
Abel model intermediate risk	79/172	Low risk	Reference	0.704 (0.643-0.765)
		Intermediate risk	5.939 (2.776-12.705)	
		High risk	14.468 (6.410-32.654)	

Abbreviations: OS, overall survival; VTT, venous tumor thrombus; PT, primary tumor; TT-GPS, VTT height, VTT Grading, Perinephric fat invasion, Sarcomatoid differentiation in PT; SSIGN, the Mayo Clinic Stage, Size, Grade and Necrosis; UISS, the University of California LosAngeles Integrated Staging System; GRANT, the GRade, Age, Nodes and Tumor; HR, hazard ratio; CI, confidential interval.

Table S10. Subgroup analysis illustrating multivariate hazard ratios and c-index for DFS according to the TT-GPS risk classification in Training cohort.

Group	Event/Total	TT-GPS risk classification	HR (95%CI)	c-index (95%CI)
SSIGN score Intermediate risk (4-7)	115/246	Low risk	Reference	0.692 (0.645-0.74)
		Intermediate risk	4.171 (2.651-6.564)	
		High risk	7.360 (4.152-13.048)	
Leibovich score Intermediate risk (3-5)	40/111	Low risk	Reference	0.7 (0.62-0.779)
		Intermediate risk	3.191 (1.639-6.213)	
		High risk	7.558 (2.706-21.108)	
UISS model Intermediate risk	107/259	Low risk	Reference	0.725 (0.68-0.77)
		Intermediate risk	4.579 (2.878-7.283)	
		High risk	9.525 (5.232-17.341)	
GRANT score unfavorable	122/208	Low risk	Reference	0.646 (0.595-0.698)
		Intermediate risk	2.808 (1.752-4.499)	
		High risk	4.987 (2.928-8.496)	
Abel model intermediate risk	100/172	Low risk	Reference	0.695 (0.641-0.749)
		Intermediate risk	4.987 (2.761-9.008)	
		High risk	11.568 (5.881-22.756)	

Abbreviations: DFS, disease-free survival; VTT, venous tumor thrombus; PT, primary tumor; TT-GPS, VTT height, VTT Grading, Perinephric fat invasion, Sarcomatoid differentiation in PT; SSIGN, the Mayo Clinic Stage, Size, Grade and Necrosis; UISS, the University of California LosAngeles Integrated Staging System; GRANT, the GRade, Age, Nodes and Tumor; HR, hazard ratio; CI, confidential interval.

Table S11. Subgroup analysis illustrating multivariate hazard ratios and c-index for OS according to the TT-GPS risk classification in China-Validation cohort.

Group	Event/Total	TT-GPS risk classification	HR (95%CI)	c-index (95%CI)
SSIGN score Intermediate risk (4-7)	88/266	Low risk	Reference	0.725 (0.672-0.778)
		Intermediate risk	5.110 (2.918-8.948)	
		High risk	9.605 (5.301-17.402)	
Leibovich score Intermediate risk (3-5)	22/124	Low risk	Reference	0.753 (0.667-0.839)
		Intermediate risk	4.099 (1.727-9.729)	
		High risk	24.284 (2.682-219.91)	
UISS model Intermediate risk	71/272	Low risk	Reference	0.751 (0.693-0.809)
		Intermediate risk	5.403 (2.854-10.227)	
		High risk	12.073 (6.163-23.651)	
GRANT score unfavorable	101/208	Low risk	Reference	0.676 (0.619-0.734)
		Intermediate risk	2.882 (1.571-5.287)	
		High risk	5.715 (3.125-10.449)	
Abel model intermediate risk	84/174	Low risk	Reference	0.669 (0.606-0.733)
		Intermediate risk	3.171 (1.583-6.352)	
		High risk	5.902 (2.893-12.040)	

Abbreviations: OS, overall survival; VTT, venous tumor thrombus; PT, primary tumor; TT-GPS, VTT height, VTT Grading, Perinephric fat invasion, Sarcomatoid differentiation in PT; SSIGN, the Mayo Clinic Stage, Size, Grade and Necrosis; UISS, the University of California LosAngeles Integrated Staging System; GRANT, the GRade, Age, Nodes and Tumor; HR, hazard ratio; CI, confidential interval.

Table S12. Subgroup analysis illustrating multivariate hazard ratios and c-index for DFS according to the TT-GPS risk classification in China-Validation cohort.

Group	Event/Total	TT-GPS risk classification	HR (95%CI)	c-index (95%CI)
SSIGN score Intermediate risk (4-7)	116/266	Low risk	Reference	0.705 (0.656-0.754)
		Intermediate risk	4.834 (3.059-7.639)	
		High risk	6.196 (3.698-10.382)	
Leibovich score Intermediate risk (3-5)	33/124	Low risk	Reference	0.751 (0.668-0.834)
		Intermediate risk	5.703 (2.795-11.635)	
		High risk	9.362 (1.172-74.808)	
UISS model Intermediate risk	104/272	Low risk	Reference	0.736 (0.687-0.785)
		Intermediate risk	5.166 (3.142-8.494)	
		High risk	8.576 (4.950-14.859)	
GRANT score unfavorable	122/208	Low risk	Reference	0.652 (0.597-0.707)
		Intermediate risk	2.394 (1.447-3.963)	
		High risk	4.161 (2.502-6.919)	
Abel model intermediate risk	107/174	Low risk	Reference	0.666 (0.613-0.719)
		Intermediate risk	4.157 (2.216-7.797)	
		High risk	6.187 (3.212-11.920)	

Abbreviations: DFS, disease-free survival; VTT, venous tumor thrombus; PT, primary tumor; TT-GPS, VTT height, VTT Grading, Perinephric fat invasion, Sarcomatoid differentiation in PT; SSIGN, the Mayo Clinic Stage, Size, Grade and Necrosis; UISS, the University of California LosAngeles Integrated Staging System; GRANT, the GRade, Age, Nodes and Tumor; HR, hazard ratio; CI, confidential interval.

Table S13. TRIPOD Checklist: Prediction model development and validation.

Section/Topic*			Checklist Item	Page
**Title and abstract**				
Title	1	D;V	Identify the study as developing and/or validating a multivariable prediction model, the target population, and the outcome to be predicted.	1
Abstract	2	D;V	Provide a summary of objectives, study design, setting, participants, sample size, predictors,outcome, statistical analysis, results, and conclusions.	4
**Introduction**				
Background and objectives	3a	D;V	Explain the medical context (including whether diagnostic or prognostic) and rationale for developing or validating the multivariable prediction model, including references to existing models.	5
	3b	D;V	Specify the objectives, including whether the study describes the development or validation of the model or both.	5
**Methods**				
Source of data	4a	D;V	Describe the study design or source of data (e.g., randomized trial, cohort, or registry data),separately for the development and validation data sets, if applicable.	11
	4b	D;V	Specify the key study dates, including start of accrual; end of accrual; and, if applicable, end of follow-up.	11
Participants	5a	D;V	Specify key elements of the study setting (e.g., primary care, secondary care, general population)including number and location of centers.	11
	5b	D;V	Describe eligibility criteria for participants.	11
	5c	D;V	Give details of treatments received, if relevant.	NA
Outcome	6a	D;V	Clearly define the outcome that is predicted by the prediction model, including how and when assessed.	12

	6b	D;V	Report any actions to blind assessment of the outcome to be predicted.	NA
Predictors	7a	D;V	Clearly define all predictors used in developing or validating the multivariable prediction model, including how and when they were measured.	12
	7b	D;V	Report any actions to blind assessment of predictors for the outcome and other predictors.	12
Sample size	8	D;V	Explain how the study size was arrived at.	13
Missing data	9	D;V	Describe how missing data were handled (e.g., complete-case analysis, single imputation, multipleimputation) with details of any imputation method.	13
Statistical analysis methods	10a	D	Describe how predictors were handled in the analyses.	11-13
	10b	D	Specify type of model, all model-building procedures (including any predictor selection), and method for internal validation.	11-13
	10c	V	For validation, describe how the predictions were calculated.	11-13
	10d	D;V	Specify all measures used to assess model performance and, if relevant, to compare multiple models.	11-13
	10e	V	Describe any model updating (e.g., recalibration) arising from the validation, if done.	NA
Risk groups	11	D;V	Provide details on how risk groups were created, if done.	11-13
Development vs. validation	12	V	For validation, identify any differences from the development data in setting, eligibility criteria, outcome, and predictors.	11-13
**Results**				
Participants	13a	D;V	Describe the flow of participants through the study, including the number of participants with and without the outcome and, if applicable, a summary of the follow-up time. A diagram may be helpful.	6
	13b	D;V	Describe the characteristics of the participants (basic demographics, clinical features, available predictors), including the number of participants with missing data for predictors and outcome.	6
	13c	V	For validation, show a comparison with the development data of the distribution of important variables (demographics, predictors and outcome).	6
Model development	14a	D	Specify the number of participants and outcome events in each analysis.	6
	14b	D	If done, report the unadjusted association between each candidate predictor and outcome.	6

Model specification	15a	D	Present the full prediction model to allow predictions for individuals (i.e., all regression coefficients, and model intercept or baseline survival at a given time point).	7-8
	15b	D	Explain how to the use the prediction model.	7-8
Model performance	16	D;V	Report performance measures (with CIs) for the prediction model.	7-8
Model-updating	17	V	If done, report the results from any model updating (i.e., model specification, model performance).	NA
**Discussion**				
Limitations	18	D;V	Discuss any limitations of the study (such as nonrepresentative sample, few events per predictor, missing data).	10
Interpretationn	19a	V	For validation, discuss the results with reference to performance in the development data, and any other validation data.	9-10
	19b	D;V	Give an overall interpretation of the results, considering objectives, limitations, results from similarstudies, and other relevant evidence.	9-10
Implications	20	D;V	Discuss the potential clinical use of the model and implications for future research.	9-10
**Other information**				
Supplementary information	21	D;V	Provide information about the availability of supplementary resources, such as study protocol, Web calculator, and data sets.	16
Funding	22	D;V	Give the source of funding and the role of the funders for the present study.	15

*Items relevant only to the development of a prediction model are denoted by D, items relating solely to a validation of a prediction model are denoted by V, and items relating to both are denoted D;V.
